# Genome-wide analysis of FRF gene family and functional identification of *HvFRF9* under drought stress in barley

**DOI:** 10.3389/fpls.2024.1347842

**Published:** 2024-01-24

**Authors:** Xiaoyan He, Yaru He, Yihuan Dong, Yu Gao, Xingcai Sun, Weiyue Chen, Xintong Xu, Congjun Su, Yifan Lv, Boyu Ren, Huayan Yin, Jianbin Zeng, Wujun Ma, Ping Mu

**Affiliations:** College of Agronomy, Qingdao Agricultural University, Qingdao, China

**Keywords:** drought stress, barley (*Hordeum vulgare* L.), *HvFRFs* gene family, *HvFRF9*, functional identification

## Abstract

FHY3 and its homologous protein FAR1 are the founding members of FRS family. They exhibited diverse and powerful physiological functions during evolution, and participated in the response to multiple abiotic stresses. FRF genes are considered to be truncated FRS family proteins. They competed with FRS for DNA binding sites to regulate gene expression. However, only few studies are available on FRF genes in plants participating in the regulation of abiotic stress. With wide adaptability and high stress-resistance, barley is an excellent candidate for the identification of stress-resistance-related genes. In this study, 22 *HvFRFs* were detected in barley using bioinformatic analysis from whole genome. According to evolution and conserved motif analysis, the 22 *HvFRFs* could be divided into subfamilies I and II. Most promoters of subfamily I members contained abscisic acid and methyl jasmonate response elements; however, a large number promoters of subfamily II contained gibberellin and salicylic acid response elements. *HvFRF9*, one of the members of subfamily II, exhibited a expression advantage in different tissues, and it was most significantly upregulated under drought stress. *In-situ* PCR revealed that *HvFRF9* is mainly expressed in the root epidermal cells, as well as xylem and phloem of roots and leaves, indicating that *HvFRF9* may be related to absorption and transportation of water and nutrients. The results of subcellular localization indicated that *HvFRF9* was mainly expressed in the nuclei of tobacco epidermal cells and protoplast of arabidopsis. Further, transgenic arabidopsis plants with *HvFRF9* overexpression were generated to verify the role of *HvFRF9* in drought resistance. Under drought stress, leaf chlorosis and wilting, MDA and O_2_^−^ contents were significantly lower, meanwhile, fresh weight, root length, PRO content, and SOD, CAT and POD activities were significantly higher in *HvFRF9*-overexpressing arabidopsis plants than in wild-type plants. Therefore, overexpression of *HvFRF9* could significantly enhance the drought resistance in arabidopsis. These results suggested that *HvFRF9* may play a key role in drought resistance in barley by increasing the absorption and transportation of water and the activity of antioxidant enzymes. This study provided a theoretical basis for drought resistance in barley and provided new genes for drought resistance breeding.

## 1 Introduction

FAR-RED ELONGATED HYPOCOTYL 3 (FHY3) and its homologous protein FAR-RED IMPAIRED RESPONSE 1 (FAR1) are important optical signaling proteins involved in the transduction of far-red light signals and are a new class of transcription factors evolved from the ancient Mutator transposases (Hudson et al., 1999; Wang and Deng, 2002). They can specifically recognize the cis-regulatory element FBS and further regulate the transcription level of multiple downstream target genes (Liu et al., 2017). FHY3/FAR1 are essentially the founding members of FAR1-RELATED SEQUENCE (FRS) family, which are conserved in terrestrial plants. Most members of the FRS family have an N-terminal C2H2 zinc finger domain (FAR1 DNA-binding domain), a central transposase domain similar to MULE transposases, and a C-end SWIM zinc finger structural domain (Lin et al., 2007). FRS-RELATED FACTOR (FRF) genes are considered to be truncated FRS family proteins because they only contain the FAR1 DNA-binding domain but not the presumed core transposase and C-end SWIM domain (Aguilar-Martínez et al., 2015). It is proposed that FRF can compete with FRS for the DNA binding sites of its targets or regulate the transcription factors of target genes by interacting with other transcription factors (Aguilar-Martínez et al., 2015). However, the underlying molecular mechanisms of this gene family need to be further studied.

The FRS and FRF families of arabidopsis (*Arabidopsis thaliana*) have 18 members, including FHY3 and FAR1, as well as 12 FRS and 4 FRF proteins (Lin and Wang, 2004; Ma and Li, 2018). Multiple sequence alignment (MSA) and conserved protein motif analysis revealed that FRS and FRF family proteins in arabidopsis can be divided into six subfamilies. Generally, most FRS and FRF family proteins have a DNA-binding domain at their N-end region. However, as an exception, *AtFRS7* and *AtFRS12* have two DNA-binding domains; *AtFRS9* has no DNA-binding domain at the N-end but only the MULE core transposase domain, and *AtFRF1*–*AtFRF4* contain only the N-end FAR1 DNA-binding domain (Ma and Li, 2018).

For the function of FRS and FRF gene families, research mainly focuses on FHY3/FAR1. Although FHY3/FAR1 are derived from transposase, they have evolved diverse and powerful physiological functions during adaptation and domestication including light signal transduction and light morphogenesis (Lin et al., 2007), chloroplast division and chlorophyll biosynthesis (Ouyang et al., 2011), circadian clock and flowering time regulation (Li et al., 2011), shoot apex meristem and flower development (Joly-Lopez et al., 2016; Li et al., 2016), starch synthesis and starch granule formation (Ma et al., 2017), abscisic acid (ABA) signaling and stress response (Tang et al., 2013), oxidative stress and plant immune response (Ma et al., 2016; Wang et al., 2016), and nutrient uptake (Ma and Li, 2021). This indicates that this class of genes is essential for plant growth, development, and stress resistance. For example, FHY3/FAR1 negatively regulated accumulation of reactive oxygen species (ROS) and oxidative-stress-induced cell death, and *fhy3* and *far1* mutant plants grew slowly and displayed severe cell death under the conditions of short days or long periods of darkness (Stirnberg et al., 2012; Ma et al., 2016; Wang et al., 2016). Loss of FHY3/FAR1 function enhanced expression of defense-response genes and thus increased the tolerance to *Pseudomonas syringae*, indicating that FHY3 and FAR1 are also involved in plant immune regulation (Wang et al., 2016). ABA plays various important roles in plant growth and development, including grain maturation, germination, and stress response (Chen K. et al., 2020). *fhy3* mutant plants were less sensitive to salt and osmotic stresses than the wild-type plants. In addition, mutant plants with disrupted FHY3/FAR1 function were less sensitive to ABA-induced stomatal motility, suggesting that FHY3/FAR1 are required for stomatal motility and drought response in plants (Tang et al., 2013). Additionally, the transcript level of *ABA INSENSITIVE5* (*ABI5*) was decreased in *fhy3*, *far1*, and *fhy3/far1* mutant plants, and after *ABI5* overexpression, the phenotype of *fhy3* mutant plants was similar to that of the wild-type plants. These results indicated that *ABI5* is regulated by FHY3 and FAR1 (Tang et al., 2013; Xu et al., 2020). Therefore, FHY3 and FAR1 bind to the *ABI5* promoter and activate its transcription, thereby mediating ABA signaling and abiotic stress responses.

Currently, there are also some research reports on FRS. Over 1000 FRS homologs have been successively predicted in various higher plant species (Lin et al., 2007; Joly-Lopez et al., 2016). *FRS10*, which was screened based on *Silver birch* genome sequencing and population genome, may play a regulatory role in the adaptability of plants to the environment (Salojärvi et al., 2017). Some predicted FRS family members may be the candidates for the regulation of panniculus and spiky degeneration in rice (Zhang et al., 2015), as well as photoperiod-dependent flowering time regulators in wheat (Kiseleva et al., 2017). However, the physiological functions and molecular mechanisms of FRF family proteins in higher plants remain largely unknown.

Drought is one of the most serious abiotic stresses affecting normal plant growth and limiting crop yield worldwide. Barley (*Hordeum vulgare* L.) is one of the oldest diploid crops in the world. It is suitable for cultivation at high altitude and cold temperature and in arid, saline–alkali, and barren land (Carter et al., 2019; Kebede et al., 2019). Therefore, it is an important plant material for exploring excellent stress-resistance genes, including drought-resistant genes. It is of great significance to clarify the drought-resistant mechanisms in barley for developing new methods to resist drought stress and improving the yield of barley and other plants. In this study, we aimed to identify drought-resistance-related FRF genes from barley. The FRF gene family was identified in barley from whole genome, and the drought response gene *HvFRF9* was screened and its function in drought resistance was verified. This study provided a theoretical basis for drought resistance in barley and provided new genes for drought resistance breeding.

## 2 Materials and methods

### 2.1 Identification of FRF genes in barley

Genomic data, CDS, protein files, and gff3 annotation files of barley Morex were downloaded from the Barley IPK database (https://galaxy-web.ipk-gatersleben.de/). The FRF sequences containing only FAR1 domain (PF03101) were searched in the Barley Protein database with a search threshold of E value < 1e^−10^ using Hmmer-search 3.0 program. The preliminary barley FRF protein sequence was submitted to SMART (http://smart.embl-heidelberg.de/), NCBI-CDD (https://www.ncbi.nlm.nih.gov/cdd/), and PFAM website (http://pfam.xfam.org/) to ensure the complete FAR1 domain structure. After removing suspicious sequences, the *HvFRF* gene family members were obtained. The protein sequences of *HvFRF* family members were uploaded to the Expasy (https://web.expasy.org) website, and their molecular weight (MW) and isoelectric point (pI) were predicted.

### 2.2 Construction of phylogenetic tree for *HvFRFs*

The FRF sequence of *A. thaliana* was downloaded from TAIR, the arabidopsis genome website (https://www.arabidopsis.org/index.jsp). The *Triticum aestivum* L. (wheat), *Brachypodium distachyon* L. (brachypodium), *Zea mays* L. (corn), *Oryza sativa* L. (rice), *Sorghum bicolor* L. (sorghum), *Glycine max* L. (soybean), and *Vitis vinifera* L. (grape) protein databases were downloaded from the Ensemble Plants website (http://plants.ensembl.org/index.html). The FRF genes containing FAR1 domain but not MULE and SWIM zinc finger structural domain were searched from wheat, brachypodium, maize, rice, sorghum, soybean, and grape protein databases using Hmmer-search 3.0 program with E value < 1e^−10^ and Blastp program based on four FRF member sequences in arabidopsis. The FRF protein sequences obtained from the preliminary screening were submitted to SMART, NCBI-CDD, and PFAM websites to further confirm the integrity of FAR1 domain and obtain FRF gene family information in the above species. MSAs were performed using Clustal W to analyze the evolutionary relationships between *HvFRF* gene family members and FRF proteins in brachypodium, soybean, grape and arabidopsis. Phylogenetic tree was constructed using Neighbor-joining method, and the bootstrap value was set to 1000 replicates. The evolutionary tree was edited using the iTOL 2.0 website (https://itol.embl.de/).

### 2.3 Chromosomal localization and gene duplication event analysis of *HvFRFs*

Tbtools software (Chen C. et al., 2020) was used to obtain the chromosome length, gene density, and chromosome location information of *HvFRF* genes from the barley genome database, and the chromosome location map of *HvFRFs* was drawn. Members of the *HvFRF* family were named according to the order of their physical positions on seven chromosomes. MCScanX software was used to analyze the gene duplication events in the barley genome and *HvFRF* family. The extracted gene collinearity results were combined with barley chromosome gene density and visualized using Tbtools to generate a collinearity map of FRF gene families within barley species.

### 2.4 Conserved motifs, gene structure, *cis*-elements and upstream IF and miRNA analysis of *HvFRFs*

MEME online tool (http://meme-suite.org/) was used to analyze the conserved motifs of HvFRF proteins, with the number of conserved motifs set as 5 and the width of motifs set as 6–150. The genetic structure of *HvFRFs* was analyzed using GSDS (http://gsds.cbi.pku.edu.cn/). The obtained files were imported into TBtools software for visualization. TBtools software was used to extract the sequence 2000-bp upstream of ATG of *HvFRF* gene family. It was submitted to PlantCARE website (http://bioinformatics.psb.ugent.be/webtools/plantcare/html/) for promoter *cis*-element analysis. The analysis results were collated and imported into TBtools for visualization. The transcription factor (IF) binding site within the promoter regions of *HvFRF* genes were predicted through the online PlantRegMap tool (http://plantregmap.gao-lab.org/binding_site_prediction.php), where the species was set to *Hordeum vulgare* L. The upstream miRNAs of *HvFRFs* were predicted using psRNATarget (https://www.zhaolab.org/psRNATarget/analysis?function=2). The main parameters were set as follows: the penalty for G:U pair was 0.5, the number of mismatches allowed in the seed region was 2, the penalty for opening gap was 2, and the penalty for extending gap was 0.5.

### 2.5 Tissue expression profile and expression characteristics of *HvFRFs* in response to drought

The expression levels of 22 *HvFRF* genes in various barley tissues such as root, epidermal cells, inflorescence, and grain at various growth stages were downloaded from WheatOmics 1.0 (http://202.194.139.32/) database. TBtools software was used to plot the heat map.

The grains of Golden Promise variety of barley with consistent grain size were selected and sterilized with 75% alcohol before germination on a filter paper. Seven days after germination, seedlings with consistent growth were selected and transferred to 5-L black plastic drums containing 1/5 Hoagland nutrient solution for further cultivation (16 h/8 h, 23°C/18°C, day/night). The nutrient solution was changed every 2–3 days during seedling growth stage, and the pH of the nutrient solution was maintained at 5.8–6.0 with continuous ventilation. When the seedlings grew to two-leaves and one-heart stage, drought stress was simulated with 15% (15 g/100 ml) PEG6000 on three biological replicates. After 1, 3, 6, 12, 24, and 48 h of drought stress treatment, the roots were cooled in liquid nitrogen and used for RNA extraction. Total RNA was extracted from the roots using SteadyPure Plant RNA Extraction Kit (Accurate Biology Co., Qingdao, China). cDNA was obtained using Evo M-MLV Reverse Transcription Kit for qPCR (Accurate Biology Co., Qingdao, China). Fluorescence quantification experiments were performed using SYBR Green Pro Taq HS Premixed type Kit (Accurate Biology Co., Qingdao, China). The PCR program used a two-step method: 95°C for 30 s, followed by 40 cycles of 95°C for 5 s and 60°C for 30 s. Relative *HvFRFs* expression values were calculated using the 2^−ΔΔCt^ relative quantification method. The primers used for qRT-PCR are given in **Supplementary Table S1**.

### 2.6 Cloning and sequence analysis of *HvFRF9*

RNA and DNA were extracted from the roots of hydroponically grown Golden Promise barley at two-leaves and one-heart stage without any treatment, and full length cDNA was generated by reverse transcription using Evo M-MLV Plus cDNA synthesis kit (Accurate Biology Co., Qingdao, China). Gene cloning primers HVFRF9-F and HVFRF9-R (**Supplementary Table S1**) were designed using Primer 5.0 to amplify the full-length open reading frame (ORF) of the *HvFRF9* gene using cDNA and DNA as templates, respectively. The amplified and purified fragments were ligated into pEASY-Blunt vector. The ligation products were used to transform competent *Escherichia coli* Trans1-T1 cells, which were cultured overnight at 37°C on LB solid medium containing 100 mg/L ampicillin (Amp). The positive clones detected using PCR were sent to Tsingke Biotech Co., Ltd. (Beijing, China) for sequencing, and the plasmids of the correctly sequenced clones were used to obtain intermediate vector pEASY-Blunt-HVFRF9.

The nucleotide sequence of *HvFRF9* gene was translated into amino acid sequences using BioXM 2.7, and the amino acid sequences were analyzed using SMART to confirm the functional domain. The HvFRF9 protein sequence was compared with that of four arabidopsis FRF members (AtFRF1, AtFRF2, AtFRF3, and AtFRF4) using DNAMAN software. The secondary structure of HvFRF9 was predicted using PSIPRED (http://bioinf.cs.ucl.ac.uk/psipred), and its three dimensional (3D) structure was builded using SWISS-MODEL (https://swissmodel.expasy.org/interactive).

### 2.7 Analysis of the expression site of *HvFRF9* using *in-situ* PCR

*In-situ* PCR of *HvFRF9* was performed according to the method described by Athman (Athman et al., 2014). From the hydroponically cultured Golden Promise, the roots and leaves at the two-leaves and one-heart stage were collected, immediately placed in fresh formaldehyde fixative (63 ml/100 ml Ethanol, 5 ml/100 ml acetic acid, 2 ml/100 ml formaldehyde). Each tissue was embedded in 5% agarose. The embedded tissue sections (60–70 μm for leaves and 50 μm for roots) were subjected to a vibrating microtome (ZQP-86, Zhixin, Shanghai, China). Each cut tissue sample was placed into a PCR tube, treated with DnaseI, and reverse transcribed for *in-situ* PCR studies. The transintron-specific primers were designed for *HvFRF9* (*in-situ*-HvFRF9-F and *in-situ*-HvFRF9-R; **Table S1**), and the gene was amplified with digoxigenin-labeled dUTP using each tissue section as a template. Anti-DIG AP antibody was added to the PCR products, incubated at 37°C for 1 h, and washed twice with the wash buffer (0.1 M Tris-HCl, 0.15 M NaCl, pH = 9.5). BM-Purple was added for staining. Further, the samples were thoroughly washed with the wash buffer and placed on slides to observe the expression site of *HvFRF9* in the roots and leaves under a positive fluorescence microscope (DM6B, Leica, Wetzlar, Germany). 18S rRNA gene was used as a positive control, and the sample without primers served as a negative control.

### 2.8 Subcellular localization of *HvFRF9*

The coding sequence of *HvFRF9* gene (stop codon removed) was amplified using the intermediate vector, pEASY-Blunt-HvFRF9, and ligated into SUPER1300-35S-GFP and P131-35S-YFP vectors, respectively. The ligation products were used to transform competent *E. coli* Trans1-T1 cells, and positive clones were obtained and sequenced. SUPER1300-35S-HvFRF9-GFP and P131-35S-HvFRF9-YFP of subcellular localization vector were obtained from the plasmids of the correctly sequenced clones (to ensure that there was no frame shift and the complete fusion protein could be translated). SUPER1300-35S-HvFRF9-GFP, nuclear localization marker, arabidopsis protoplasts, and PEG4000 solution were mixed and placed in water bath for 15 min. Protoplasts were collected after washing with W5 solution (154 mM NaCl, 125 mM CaCl_2_, 5 mM KCl, 5 mM glucose, and 2 mM MES). The fluorescence distribution was observed under a laser confocal microscope (A1 HD25, Nikon, Tokyo, Japan). P131-35S-HvFRF9-YFP plasmid was transferred into *Agrobacterium tumefaciens* EHA105, which was used to infect tobacco (*Nicotiana benthamiana*) at the 4–5 leaf stage. Between 36 and 72 h after infection, undamaged tobacco leaves were selected to make sample slides. The fluorescence distribution was observed under a laser confocal microscope (A1 HD25, Nikon, Tokyo, Japan). The culture conditions for tobacco were 24°C/26°C, 16 h/8 h, day/night.

### 2.9 Establishment of overexpression carrier of *HvFRF9* and genetic transformation of arabidopsis

The overexpression primers HvFRF9-OE-F and HvFRF9-OE-R (**Supplementary Table S1**) were designed using Primer 5.0, and the coding sequence (with stop codon) of *HvFRF9* gene was amplified from the intermediate vector pEASY-Blunt-HvFRF9 and connected to the SUPER1300 vector. The ligation product was used to transform competent *E. coli* Trans1-T1 cells. Positive clones were obtained and sequenced. The cloned plasmids with correct sequencing were used to obtain the overexpression carrier SUPER1300-35S-HvFRF9. This recombinant plasmid was used to transform *A. tumefaciens* GV3101. The transformed bacterial cells were grown in liquid YEB medium containing rifampicin (20 mg/L) and kanamycin (50 mg/L) on a shaker till OD_600_ of 1.2 was obtained. The cells were collected by centrifugation at 5000 g and resuspended in osmotic buffer (5 g/100 mL sucrose, 20 μL/100 mL Silwet L-77, 0.05 g/100 mL 1/2 MS) to obtain the inoculum for infection with the OD_600_ of 0.8–1.0. The flower bud parts of wild-type arabidopsis (Col-0) were inoculated with the inoculum for infection, and the seeds of the T1 generation were harvested when arabidopsis plants were mature. The seeds of T1 generation were disinfected and seeded in solid medium containing 25 mg/L hygromycin, from which the seedlings with developed roots and strong growth were selected for further cultivation. The seeds of T2 generation were harvested from each plant, and These seeds were further grown on culture medium with 25 mg/L hygromycin, and strains with a 3:1 separation ratio between positive and negative seedlings were selected to verify the expression level of *HvFRF9* gene. The primer information is given in **Supplementary Table S1**. Three lines with high expression levels were selected, and T3 grains were harvested from each plant to obtain the *HvFRF9* overexpression lines. *Arabidopsis* culture conditions were as follows: 23°C, 16 h/8 h, day/night.

### 2.10 Identification of drought-resistant phenotypes of arabidopsis with *HvFRF9* overexpression

Wild-type and *HvFRF9*-overexpressing arabidopsis grains were sterilized and planted on 1/2 MS solid medium without antibiotics. After 3 days of vernalization, the medium was placed in a light incubator (23°C, 16 h/8 h, day/night). After 1 week, the seedlings with consistent growth were selected and transferred to MS solid medium containing various concentrations of mannitol (0, 50, 100, and 200 mM). Three biological replicates were used for each treatment. After 1 week, the main root length of arabidopsis was observed and measured, and the fresh weight of each line was determined.

Wild-type and *HvFRF9*-overexpressing arabidopsis seedlings grown normally for 1 week on 1/2 MS solid medium were transferred to 0.2-L seedling pots for continued cultivation (23°C, 16 h/8 h, day/night), each containing nutrient soil of equal weight and humidity. Drought treatment was performed as follows after 3 days of culture. The control group was watered normally (keeping the soil water content at 80% ± 5%), and the drought group did not receive water. Three biological replicates with six plants per replicate were used in the control and drought groups. After 14 days of drought treatment, the phenotypes of arabidopsis were observed, and the physiological parameters were measured. The superoxide anion (O_2_^−^) activity in the leaves of arabidopsis was measured using NBT method (Bournonville and Diaz-Ricci, 2011). Malondialdehyde (MDA) and proline (PRO) contents in the leaves were determined using MDA and PRO Content Assay Kits (Solarbio Science & Technology Co., Ltd., Beijing, China), respectively. Catalase (CAT), superoxide dismutase (SOD), and peroxidase (POD) activities in the leaves were determined using CAT assay kit (Visible light), SOD assay kit (WST-1 method), and POD assay kit (Nanjing Jiancheng Bioengineerring Institute, Nanjing, China), respectively.

### 2.11 Data analysis

IBM SSPS Statistics 22 software was used for the statistical analysis of data. The significance of difference (p < 0.05) was tested by least-significant difference using one-way ANOVA, and the significance of data was marked by Waller–Duncan letter labeling method (p < 0.05).

## 3 Results

### 3.1 Identification of FRF genes in barley and analysis of their physicochemical properties

A total of 52 candidate FAR1-domain-containing genes were identified in the genome-wide data of barley. In total, 22 FRF family proteins were identified using SMART, NCBI-CDD, and PFAM. The 22 *HvFRFs* were named *HvFRF1–HvFRF22* (**Table 1**) based on their positional order on barley chromosomes. The physicochemical properties of HvFRF proteins were analyzed. The number of amino acids ranged from 189–399 aa, with an average of 240.9 aa. The MW ranged from 21.6–45.7 kDa, with an average of 27.9 kDa. The pI ranged from 5.09–10.67, with an average of 8.83. Moreover, 86% of the proteins had pI > 7, indicating that the proteins in this family were slightly alkaline (**Table 1**).

**Table 1:** Basic information of *HvFRF* gene family in barley.

Gene name	Gene ID	ORF^a^ (aa)	MW^b^ (kD)	pI^c^
*HvFRF1*	HORVU.MOREX.r3.2HG0109890.1	242	27.92	7.71
*HvFRF2*	HORVU.MOREX.r3.2HG0192790.1	216	25.39	9.87
*HvFRF3*	HORVU.MOREX.r3.3HG0290700.1	230	26.63	9.59
*HvFRF4*	HORVU.MOREX.r3.4HG0343370.1	212	24.49	9.85
*HvFRF5*	HORVU.MOREX.r3.4HG0363560.1	269	30.45	9.03
*HvFRF6*	HORVU.MOREX.r3.4HG0405770.1	236	27.31	8.68
*HvFRF7*	HORVU.MOREX.r3.4HG0410460.1	225	26.67	9.11
*HvFRF8*	HORVU.MOREX.r3.5HG0442590.1	199	23.07	8.11
*HvFRF9*	HORVU.MOREX.r3.5HG0479800.1	263	31.00	9.39
*HvFRF10*	HORVU.MOREX.r3.5HG0496120.1	213	24.90	10.67
*HvFRF11*	HORVU.MOREX.r3.5HG0507800.1	249	28.95	10.22
*HvFRF12*	HORVU.MOREX.r3.5HG0516020.1	253	29.78	8.01
*HvFRF13*	HORVU.MOREX.r3.5HG0523890.1	265	30.70	9.66
*HvFRF14*	HORVU.MOREX.r3.5HG0537560.1	249	29.11	9.12
*HvFRF15*	HORVU.MOREX.r3.6HG0553570.1	200	21.67	7.34
*HvFRF16*	HORVU.MOREX.r3.6HG0597910.1	189	21.60	8.57
*HvFRF17*	HORVU.MOREX.r3.6HG0626760.1	254	29.58	9.21
*HvFRF18*	HORVU.MOREX.r3.7HG0674170.1	203	23.24	9.64
*HvFRF19*	HORVU.MOREX.r3.7HG0688260.1	222	26.21	9.27
*HvFRF20*	HORVU.MOREX.r3.7HG0700660.1	248	28.58	6.79
*HvFRF21*	HORVU.MOREX.r3.7HG0733350.1	399	45.73	5.09
*HvFRF22*	HORVU.MOREX.r3.7HG0738600.1	263	31.07	9.28

a : ORF is the abbreviation of Open Reading Frame.

b : MW is the abbreviation of Molecular Weight.

c : pI is the abbreviation of Isoelectric Point.

### 3.2 Phylogenetic tree of *HvFRF* gene family

To screen and identify FRF genes in the genomes of monocotyledonous grasses such as wheat, brachypodium, corn, rice, and sorghum and dicotyledonous plants such as soybean and grape, four FRF (FRF1–FRF4) protein sequences in *Arabidopsis* were used. The FRF genes containing only the FAR1 domain but not the MULE domain and SWIM zinc finger structural domain were searched from the protein databases of the seven species mentioned above using the Hmmer-search 3.0 and the Blastp programs. In total, 17 *BdFRF*, 21 *GmFRF* and 11 *VvFRF* genes were identified from brachypodium, soybean and grape genomes, respectively. It can be seen that the number of FRF genes in barley was almost the same, twice, and 5 times that of FRF genes in soybean, brachypodium, grape, and arabidopsis, respectively. Nevertheless, FRF members were not found in the genomes of wheat, corn, rice, and sorghum, which are the grasses similar to barley.

**Figure 1:**
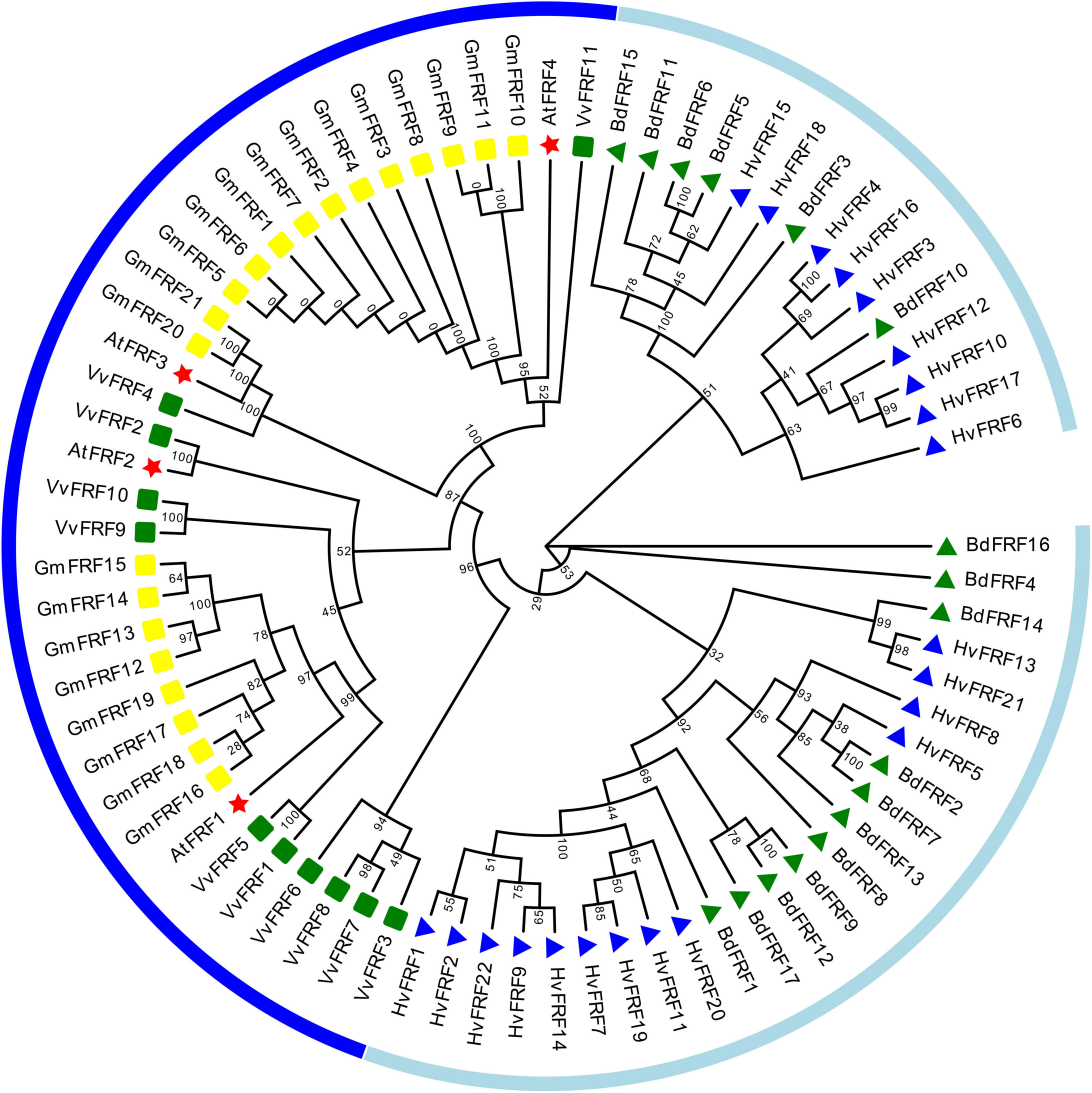
Phylogenetic trees of FRF gene families in barley, brachypodium, soybean, grape, and arabidopsis. Multiple sequence alignments were performed with MEGA-11, and phylogenetic trees were constructed using the Neighbor-joining method. Light blue lines indicate the branch where the monocotyledonous plants (barley and brachypodium) FRF genes are located, and dark blue lines indicate the branch where the dicotyledonous plants (arabidopsis, soybean and grape) FRF genes are located. Blue triangles represent *HvFRFs*; green triangles represent *BdFRFs*; yellow squares represent *GmFRFs*; green squares represent *VvFRFs*, and red pentagrams represent *AtFRFs*.

The protein sequences of 22 barley *HvFRF* genes, 17 brachypodium *BdFRF* genes, 21 soybean *GmFRF* genes, 11 grape *VvFRF* genes, and 4 arabidopsis *AtFRF* genes that were screened were used to construct a phylogenetic tree (**Figure 1**). The results revealed that the FRF genes of the dicotyledonous plants such as soybean, arabidopsis, and grape were more closely related, whereas those of the monocotyledon grass crops barley and brachypodium were separated into a large branch. This indicated that the homology of FRF gene sequences between barley and brachypodium is higher than that of dicotyledonous plants.

### 3.3 Chromosomal localization and gene duplication events of *HvFRF* genes

**Figure 2:**
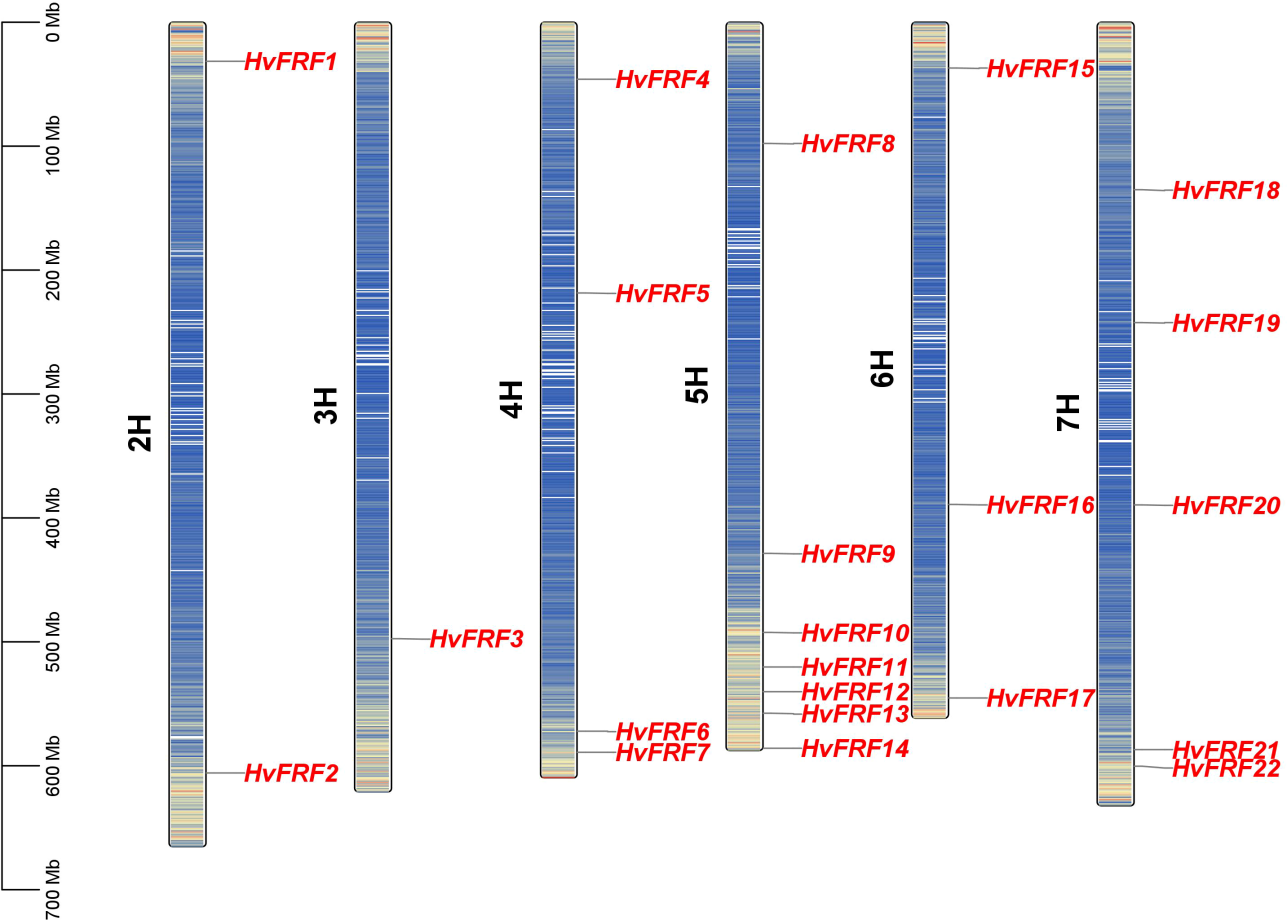
Chromosome localization of *HvFRFs*. The left bar indicates the length and physical position of the chromosome. The color of the bands on the chromosome represents gene density, where red and blue indicate regions of high and low gene densities, respectively.

The 22 *HvFRF* genes were unevenly distributed on the seven chromosomes of barley (**Figure 2**). The most densely distributed *HvFRF* genes were found on chromosomes 5H and 7H (7 and 5, respectively). The numbers of *HvFRF* genes on chromosomes 4H, 6H, 2H, and 3H were 4, 3, 2, and 1, respectively, whereas no *HvFRF* gene was found on chromosome 1H.

To further analyze the evolutionary relationship of FRF genes in barley, the repeated events of 22 *HvFRF* genes in barley were analyzed. The results revealed no collinearity gene pair among the 22 *HvFRF* genes in barley, indicating that no gene duplication event occurred in the *HvFRF* gene family (**Supplementary Figure S1**).

### 3.4 Conserved motifs and gene structures of *HvFRFs*

**Figure 3:**
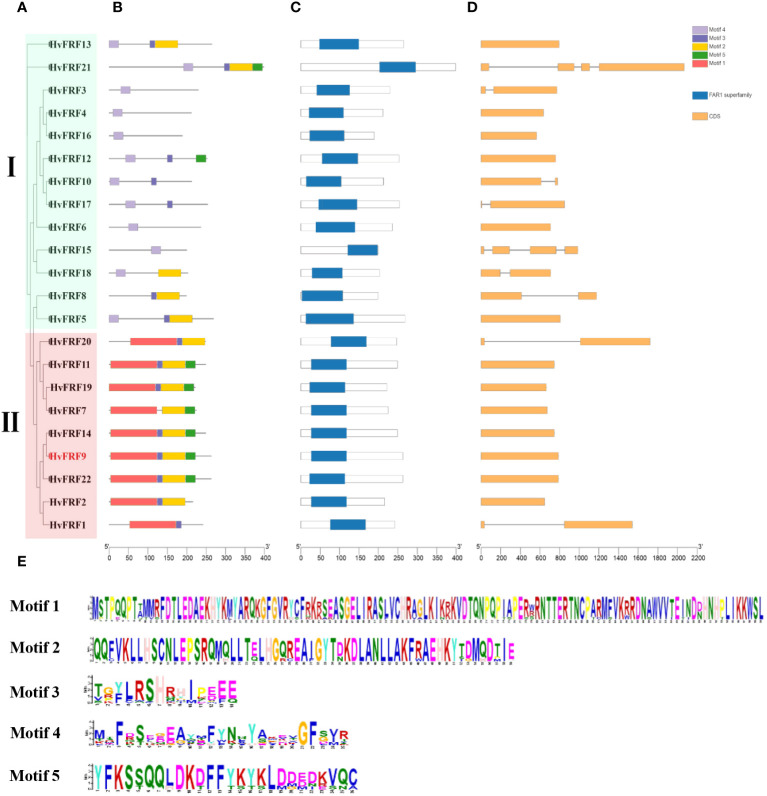
Sequence characteristics of *HvFRF* genes. **(A)** Phylogenetic tree of *HvFRF* gene family; **(B)** Conserved motifs in HvFRF proteins; **(C)** Core domain of HvFRF proteins; **(D)** *HvFRF* gene structure; **(E)** Logo diagram of the five conserved motifs of HvFRF proteins.

To understand the sequence characteristics of the 22 *HvFRF* genes in barley, the conserved motifs (**Figures 3B, E**), core domains (**Figure 3C**), and gene structure characteristics (**Figure 3D**) were analyzed based on the evolutionary analysis (**Figure 3A**). The core domain analysis results revealed that all HvFRFs had one FAR1 domain (**Figure 3C**). The results of conserved motif analysis revealed that the HvFRF proteins of the same subfamily had similar motif composition (**Figure 3B**). Among them, 12 members of 13 HvFRF proteins of subfamily I contained conserved Motif 4, and each of the nine HvFRF proteins of subfamily II contained a long and conserved Motif 1. Subfamily II contained Motif 2, Motif 3, and Motif 5, accounting for 89%, 89%, and 67% of the members, respectively. The members of subfamily I contained Motif 2, Motif 3, and Motif 5, accounting for only 38%, 54%, and 15%, respectively. The members of subfamily II contained more than three different motifs accounting for 89%, whereas the members of subfamily I only accounted for 31%. It can be seen that compared with HvFRFs in subfamily I, subfamily II members had more types and numbers of Motifs, and the Motif composition was more regular. Gene structure analysis revealed that more than half of the *HvFRF* genes from subfamily I contained 1–3 introns; however, only 2 members of the 9 *HvFRF* genes from subfamily II contained one intron, and the remaining members had no introns (**Figure 3D**). These results suggested that the *HvFRFs* of subfamily II have a simpler gene structure.

### 3.5 *Cis*-element in the promoter of *HvFRFs*, transcription factor-HvFRF and miRNA-HvFRF regulatory networks

**Figure 4:**
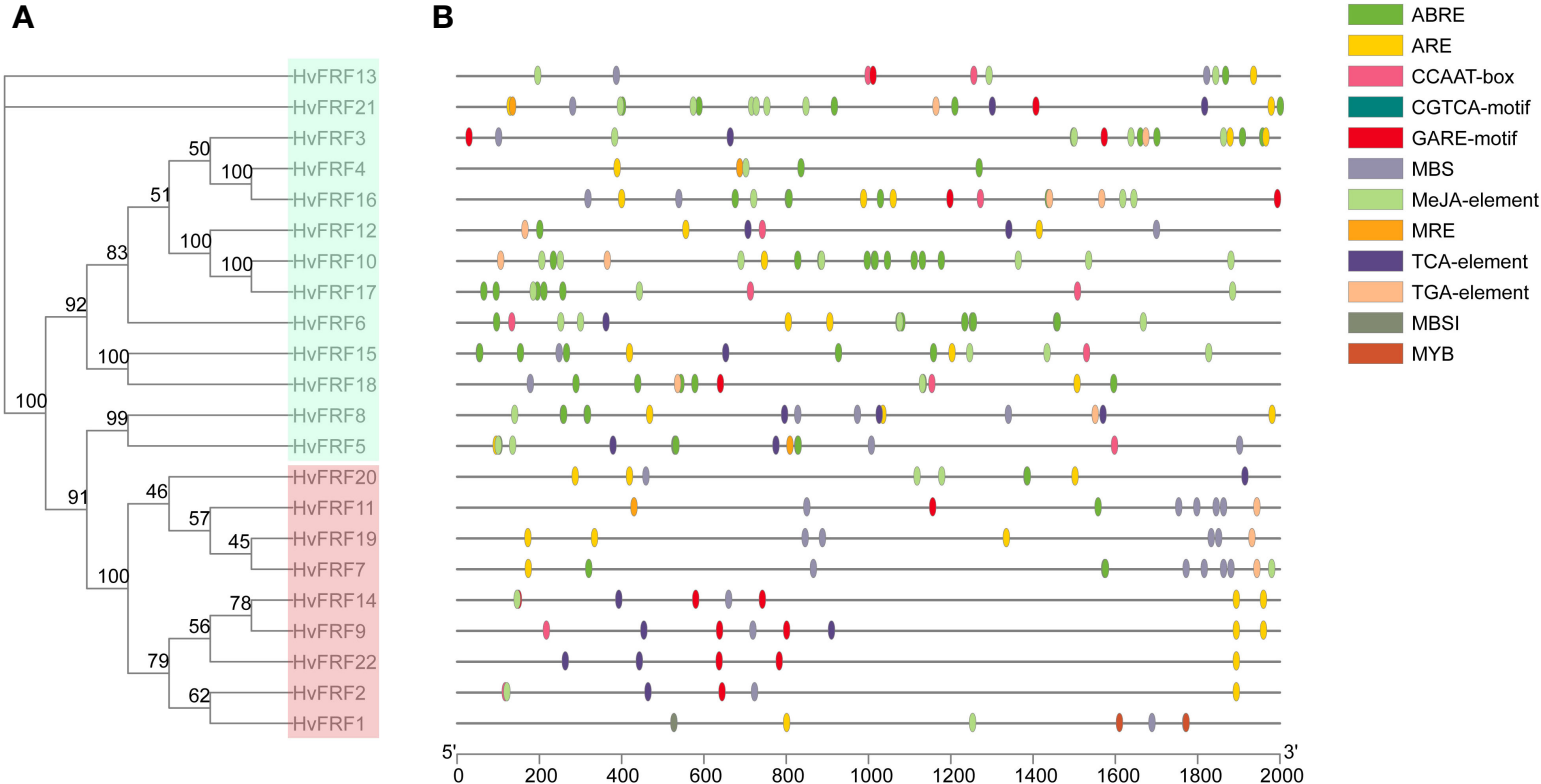
*Cis*-element in the promoter of *HvFRFs*. **(A)** Phylogenetic tree of *HvFRF* gene family. **(B)** Schematic of *cis*-element in the promoter of *HvFRFs*.

To understand the role of *HvFRFs* in stress response and signal transduction pathways in barley, the *cis*-elements in the 2000-bp promoter zone upstream of ATG of 22 *HvFRF* genes were analyzed using the PlantCARE website. The results revealed that the promoter zone of *HvFRF* genes contained a series of important hormone- and abiotic-stress-response elements, including ABRE, ARE, GARE, MBS, MRE, TCA-element, TGA-element, and MeJA-element (**Figure 4**) involved in ABA response, anaerobic induction regulation, gibberellin response, drought response, light response, salicylic acid response, auxin response, and methyl jasmonate response, respectively. Importantly, the composition and distribution of *cis*-elements in the promoter zone of *HvFRF* genes were clearly different between the two subfamilies. *HvFRFs* from subfamily I contained a large number of ABA and methyl jasmonate response elements; however, most *HvFRFs* from subfamily II contained gibberellin and salicylic acid response elements, and the distribution characteristics of the elements were similar. Additionally, except for *HvFRF4*, *HvFRF6*, *HvFRF10*, and *HvFRF17*, the promoter zones of other *HvFRF* genes contained essential element MBS in response to drought stress. *HvFRF2*, *HvFRF9*, and *HvFRF14* genes from subfamily II simultaneously contained gibberellin, salicylic acid, and drought response elements.

To understand the regulation networks of transcription factors on *HvFRFs*, The upstream transcription factors of *HvFRF* genes were predicted. 4181 binding sites of 33 kinds of transcription factors are identified within the promoter regions of 22 *HvFRF* genes, among which, ERF and C2H2 are the two main types, with a binding site of 955 and 444, respectively (**Supplementary Table S2**). To determine whether a miRNA-HvFRFs regulatory network exists in barley, 71 known miRNAs were scanned. Finally, 3 miRNAs (*hvu-miR5052*, *hvu-miR6199* and *hvu-miR6200*) were predicted to inhibit the transcription level of HvFRFs (*HvFRF16*, *HvFRF8* and *HvFRF10*) (**Supplementary Table S3**).

### 3.6 Tissue expression characteristics and expression patterns of *HvFRF* genes in response to drought stress

**Figure 5:**
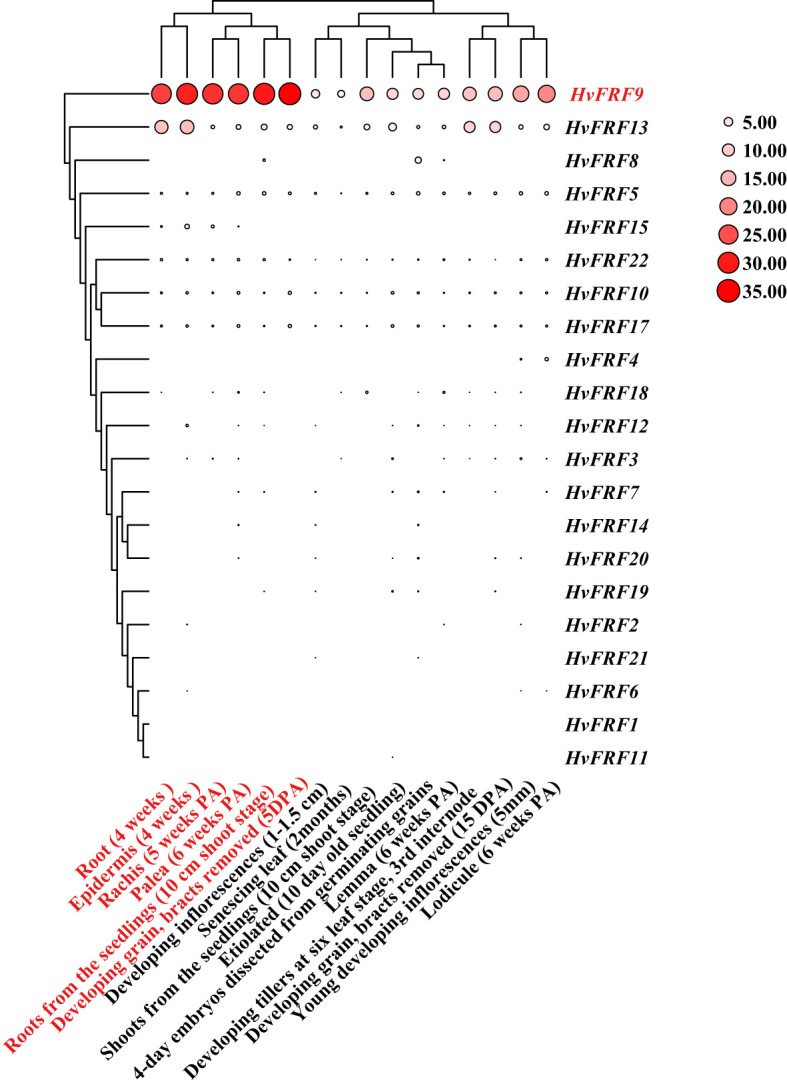
Heat map of tissue expression of *HvFRF* genes. The larger the circle and redder the color, the higher the expression. PA, post-anthesis; DPA, day post-anthesis.

To understand the expression of *HvFRF* genes in various developmental stages and various tissues of barley, the expression data of Morex varieties were downloaded from WheatOmics 1.0 and a heat map was plotted (**Figure 5**). The results revealed that the expression levels of *HvFRF* genes were significantly different in the root, epidermal cells, leaves, inflorescence, and grain at various developmental stages. Most *HvFRF* genes were expressed at low levels or even not expressed in all tissues. *HvFRF9* gene was highly expressed in various tissues, particularly in the root, epidermis, rachis, palea, and developing grain.

**Figure 6:**
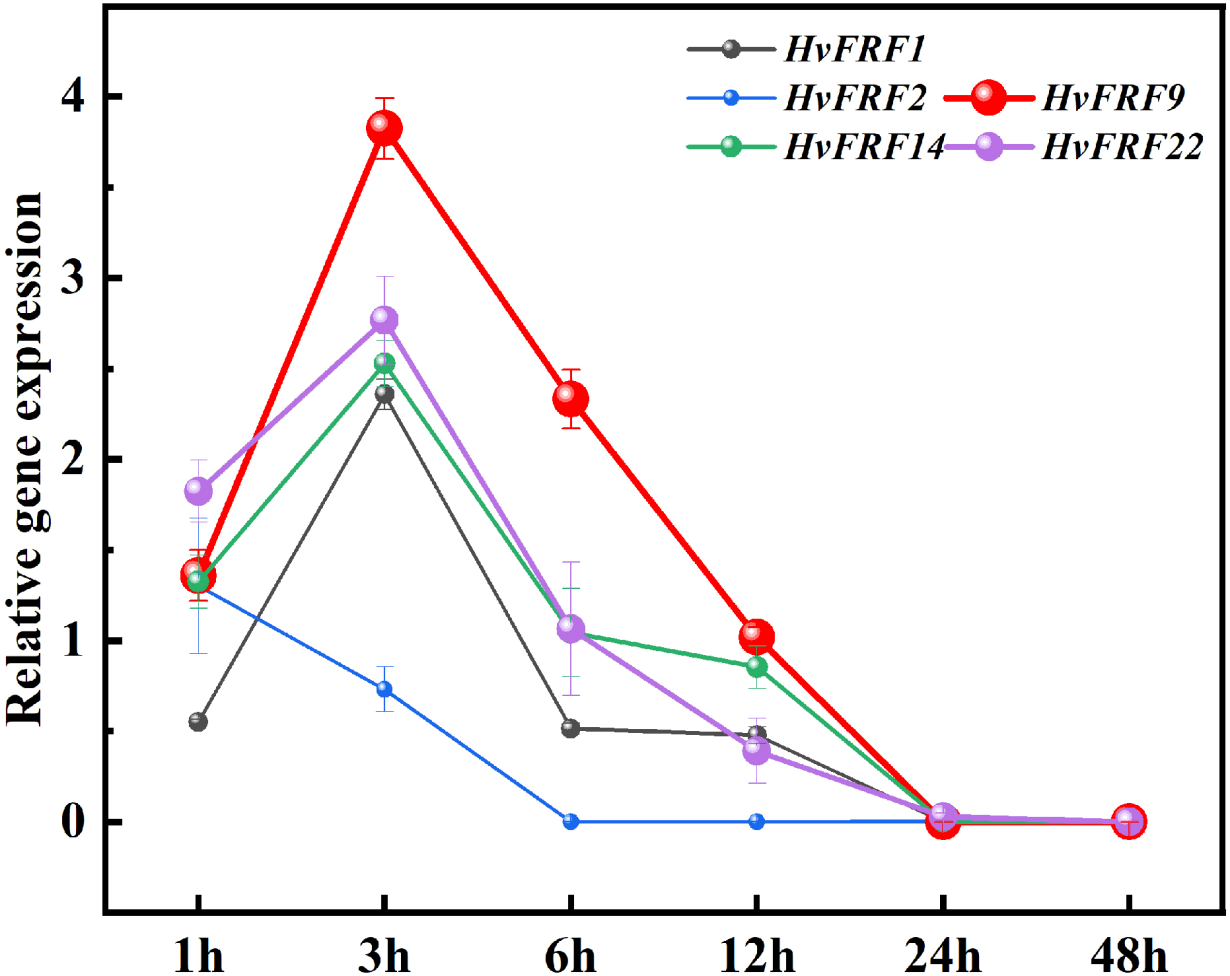
Expression patterns of *HvFRF1*, *HvFRF2*, *HvFRF9*, *HvFRF14*, and *HvFRF22* in response to drought stress.

Abiotic stress is an important adverse factor that limits crop growth and grain yield, and drought is one of the main factors. To further study the expression characteristics of *HvFRF* genes under drought resistance, an analysis was conducted on the expression in response to drought stress of *HvFRF9* with tissue expression advantages, as well as *HvFRF1*, *HvFRF2*, *HvFRF14*, and *HvFRF22* genes in the same evolutionary branch as *HvFRF9*. The results revealed that all five genes were upregulated in PEG-treated roots of Golden promise barley in response to drought stress. Except for *HvFRF2* gene, the expression levels of the other four genes reached the maximum at 3 h of drought treatment; however, *HvFRF9* gene was the most significantly upregulated (3.9 times higher than the control) (**Figure 6**). With the extension of PEG-treatment time, the expression level of the five genes decreased and returned to that of the control at 24 h of treatment (**Figure 6**). According to the above results, *HvFRF9* was upregulated most significantly in response to drought stress, and it was speculated that *HvFRF9* was closely related to drought resistance in barley.

### 3.7 Cloning and expression localization of *HvFRF9* in barley

The ORF of *HvFRF9* gene was amplified using the DNA and cDNA from Golden promise roots as templates, and agarose gel electrophoresis revealed that the amplified products of DNA and cDNA were of the same size (**Supplementary Figure S2A**). The amplified products were ligated into the pEASY-Blunt vector and sequenced. The results revealed that the 789-bp ORF of *HvFRF9* was intron-free and encoded a 262-aa protein sequence (**Supplementary Figure S2B**). Alignment of HvFRF9 protein sequence with arabidopsis AtFRF1, AtFRF2, AtFRFR3, and AtFRF4 protein sequences revealed that the above proteins contained a FAR1 conserved domain (**Supplementary Figure S3A**). The highly conserved amino acid residues among the five proteins were consistent with the HMM logo map of the FAR1 domain in the PFAM website (**Supplementary Figure S3B**). Secondary structure (**Supplementary Figure S4A**) and 3D (**Supplementary Figure S4B**) model prediction revealed that there were 7 helix and 9 strand in HvFRF9 protein sequence.

**Figure 7:**
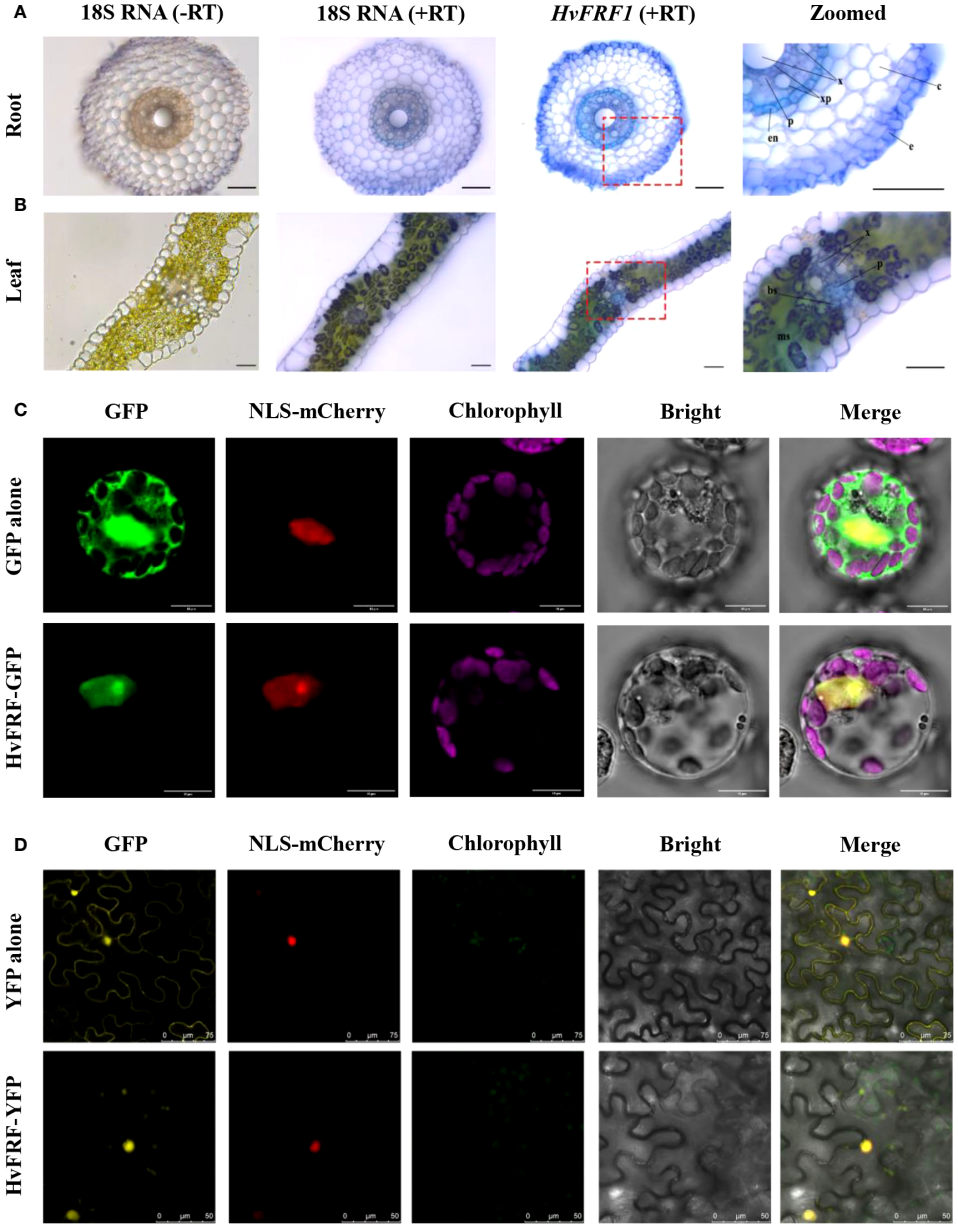
Localization of *HvFRF9.* Tissue sections of the *HvFRF9* gene in barley roots and leaves under *in-situ* PCR are shown in **(A, B)**, respectively. The 18S RNA amplification product was used as a positive control, and the sample without primers was used as a negative control. The two images on the far right are magnified images of the area in the red box. e, epidermis; c, cortical parenchyma cells; x, xylem; p, phloem, en, endodermis; bs, bundle sheath; ms, mesophyll. Bar = 100 μm. **(C, D)** indicate the subcellular localization of *HvFRF9* in arabidopsis protoplasts and tobacco epidermal cells, respectively. GFP: green fluorescence channel, YFP: yellow fluorescence channel, NLS-mCherry: nuclear localization marker, Chlorophyll: chloroplast autofluorescence, Bright: light field, Merge: superposition of fluorescence and light field.

*In-situ* PCR was performed using the roots and leaves of Golden promise at the two-leaves and one-heart stage under hydroponic condition, and the staining of tissue sections was observed under a positive fluorescence microscope (DM6B, Leica, Wetzlar, Germany). In the roots, *HvFRF9* transcripts were mainly detected in the phloem, xylem, and epidermal cells (**Figure 7A**); in the leaves, they were mainly detected in the phloem, xylem, and bundle sheath (**Figure 7B**). Therefore, *HvFRF9* may be related to the absorption and transport of water and mineral nutrients in barley.

The subcellular localization vector of *HvFRF9* was used to transform arabidopsis protoplasts and four-leaf tobacco using PEG and *Agrobacterium*-mediated methods, respectively. The subcellular localization of *HvFRF9* was observed under a laser confocal microscope (A1 HD25, Nikon, Tokyo, Japan). The results revealed that *HvFRF9* was localized only in the nucleus of arabidopsis protoplasts (**Figure 7C**). In tobacco epidermal cells, the gene was mainly located in the nucleus, and a small amount was also expressed in the chloroplast (**Figure 7D**). It can be concluded that *HvFRF9* mainly functions in the nucleus and is a transcription factor.

### 3.8 Determination of the role of *HvFRF9* in drought resistance via its overexpression in arabidopsis

**Figure 8:**
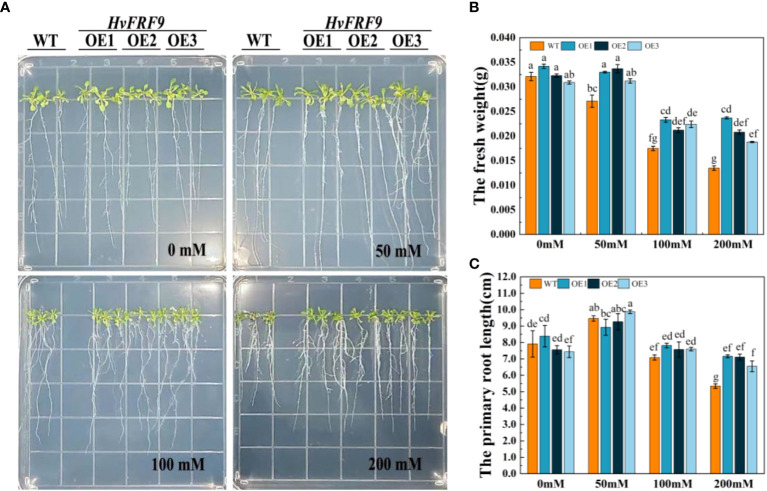
Effects of treatment with various concentrations of mannitol on *HvFRF9*-overexpressed and wild-type arabidopsis. **(A)** Growth phenotypes, **(B)** fresh weight, and **(C)** primary root length of *HvFRF9*-overexpressed and wild-type plants under various mannitol concentrations. Data are the means of three biological replicates. The different lowercase letters represent significant differences at the 5% level.

To investigate the effects of drought stress on the root growth of *HvFRF9*-overexpressed and wild-type arabidopsis, arabidopsis seedlings were treated with 0, 50, 100, or 200 mM mannitol. No significant difference in root length and fresh weight was observed between wild-type and transgenic arabidopsis grown in MS medium without mannitol. Treatment with 50 mM mannitol slightly promoted the root length of wild-type and transgenic arabidopsis but inhibited the increase in the fresh weight of wild-type plants. With the increase of mannitol concentration, the inhibition of root growth of arabidopsis gradually increased; however, the degree of inhibition was significantly less in transgenic arabidopsis than in wild-type arabidopsis (**Figure 8**). The most obvious difference between the wild-type and transgenic lines was observed under the treatment of 200 mM mannitol; transgenic arabidopsis plants exhibited almost no wilting and yellowing of leaves, longer root systems, and more lateral roots than the wild-type plants (**Figure 8A**). Specifically, the average fresh weight of transgenic lines OE1, OE2, and OE3 was 0.0237, 0.0208, and 0.0188 g, respectively, whereas that of wild-type plants was only 0.0135 g. The fresh weight of transgenic lines was significantly higher than that of wild-type plants (**Figure 8B**). Moreover, the average root length of transgenic lines OE1, OE2, and OE3 (7.24, 7.07, and 6.27 cm, respectively) was significantly higher than that of wild-type plants (5.15 cm) (**Figure 8C**). These results indicated that *HvFRF9* overexpression lines exhibited better resistance to drought stress under mannitol treatment than wild-type arabidopsis.

**Figure 9:**
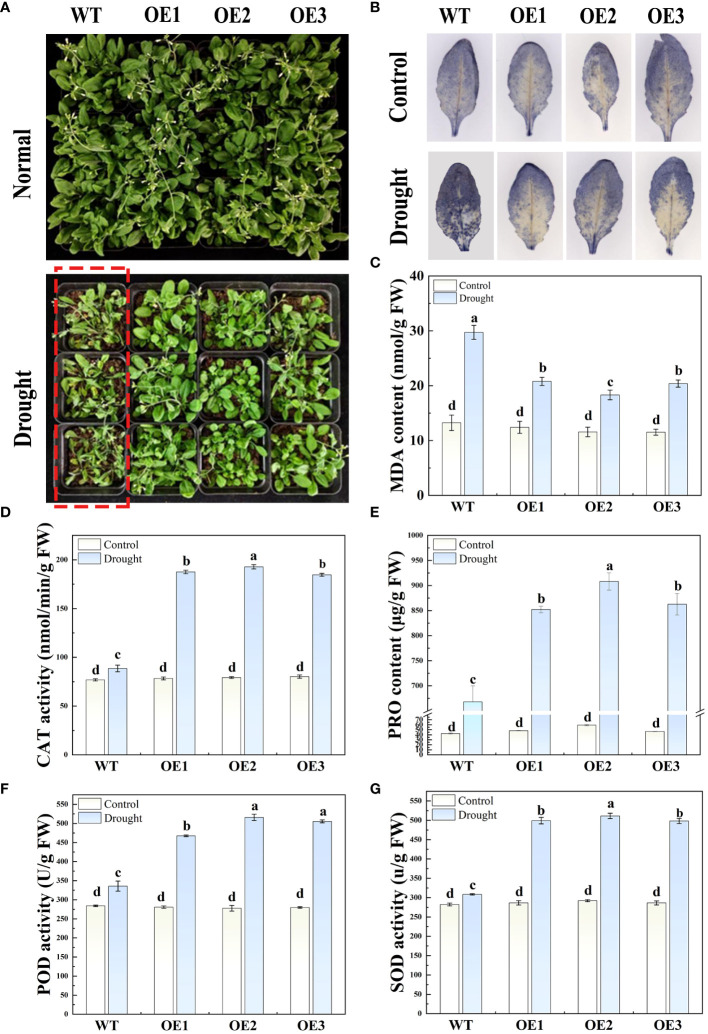
The phenotypes and various physiological indices in response to drought stress in *HvFRF9*-overexpressed and wild-type arabidopsis were determined. **(A)** Growth phenotype, **(B)** O_2_^−^ accumulation, **(C)** MDA content, **(D)** CAT activity, **(E)** PRO content, **(F)** POD activity, and **(G)** SOD activity in *HvFRF9-*overexpressed and wild-type arabidopsis under normal watering and drought treatment conditions. Data are the means of three biological replicates. The different lowercase letters represent significant differences at the 5% level.

To further verify the role of *HvFRF9* in drought resistance, a pot experiment was conducted to observe the phenotypes of *HvFRF9*-overexpressed and wild-type arabidopsis in response to drought stress, and the related physiological parameters were measured. No significant difference in terms of shoot growth was observed between *HvFRF9*-overexpressed and wild-type arabidopsis under normal watering condition. Under drought treatment, the growth of both overexpressed and wild-type plants was inhibited to a certain extent; however, the leaves of wild-type plants were severely wilted, turned yellow, and even fell off, whereas the *HvFRF9* overexpression lines could still grow normally (**Figure 9A**). This indicated that in the *HvFRF9* overexpression lines, growth was significantly less inhibited than in the wild-type plants under drought conditions. Additionally, drought treatment promoted the accumulation of O_2_^−^ in the leaves compared with the normally watered plants; however, the accumulation of O_2_^−^ was significantly lower in the leaves of the *HvFRF9* overexpression lines than in those of the wild-type plants (**Figure 9B**). Under normal watering conditions, no significant difference was observed in terms of MDA content in the leaves between *HvFRF9*-overexpressed and wild-type arabidopsis. Drought treatment increased the MDA content; however, the increase in MDA in the leaves of *HvFRF9* overexpression lines was significantly smaller than that in the leaves of wild-type plants (**Figure 9C**). Additionally, drought treatment increased CAT activity (**Figure 9D**), PRO content (**Figure 9E**), POD activity (**Figure 9F**), and SOD activity (**Figure 9G**) in the leaves compared with normal watering. However, CAT activity (**Figure 9D**), PRO content (**Figure 9E**), POD activity (**Figure 9F**), and SOD activity (**Figure 9G**) in the leaves of *HvFRF9* overexpression lines were significantly higher than those in the leaves of wild-type plants. These results suggested that *HvFRF9* overexpression could enhance the drought resistance of transgenic arabidopsis by increasing the antioxidant enzyme activity and reducing the increase in O_2_^−^ and MDA contents.

## 4 Discussion

Drought is one of the important abiotic stress factors limiting crop growth and yield. Improving the drought-resistant ability of crops is of great significance. Exploring the drought-resistant genes and their function and mechanism is an important basis for developing new drought-resistant crops. Barley exhibits great adaptation and strong stress resistance characteristics. Therefore, it is an excellent material for exploring drought-resistance-related genes. FRS and FRF gene families are novel transcription factors derived from transposases, which play important roles in various life processes in plants such as light signal response, growth response, and stress response by regulating the expression of downstream genes (Ma et al., 2016). However, current studies on these genes are mainly focused on the model plant arabidopsis. To explore the evolution and function of FRF gene family in barley, 22 FRF genes in barley were identified from genome-wide analysis (**Table 1**), and *HvFRF9* was identified to have drought resistance function. The discovery of this gene can provide a new gene resource for genetic improvement of drought resistance in plants.

### 4.1 FRS and FRF gene families involved in the regulation of drought resistance and other environmental adaptability in plants

*Medicago ruthenica* (L.) *Trautv*. is a wild perennial legume forage widely distributed in semiarid grasslands with outstanding tolerance to environmental stresses (Yoder et al., 2013). Genome sequencing and genetic studies have reported that the expansion of the FHY3/FAR1 family in the wild *M. ruthenica* played an important role in drought stress tolerance compared with the cultivated alfalfa (Wang et al., 2021). It has been demonstrated that FHY3/FAR1 in arabidopsis is a positive regulator of ABA signaling and can exhibit increased expression in response to ABA and abiotic stresses. Compared with the wild type, *fhy3* and *far1* mutants exhibit wider stomata and faster water loss and are more sensitive to drought (Li et al., 2016). Myo-Inositol-1-phosphate synthase (MIPS) is a rate-limiting enzyme that catalyzes the biosynthesis of inositol and plays a crucial role in plant growth and development. In arabidopsis, FHY3/FAR1 directly binds to the promoter of *MIPS1* and activates its expression, thereby promoting inositol biosynthesis and preventing light-induced oxidative stress and SA-dependent cell death (Joly-Lopez et al., 2016). Abiotic stresses, particularly salt and temperature stresses, are important environmental factors affecting wood productivity. In *Eucalyptus grandis* W. Hill ex Maiden, FHY3/FAR1 family genes play a role in the response to salt and temperature treatments, and these genes may be used in improving wood yield of *E*. *grandis* (Dai et al., 2022). FHY3 can interact with *MYC2*, a key transcription regulator of jasmonic acid response, and coregulate the expression of jasmonic-acid-responsive defense genes (Liu et al., 2019). In *Camellia sinensis* (L.) O. Kuntze, most *CsFHY3/FAR1s* are upregulated in response to drought stress. Genetic and molecular evidence suggested that FRS7 and FRS12 interact with NOVEL INTERACTOR OF JAZ (NINJA) to regulate glucosinolate-biosynthesis-related genes and participate in the defense mechanism of arabidopsis (Fernández-Calvo et al., 2020). Expression analysis revealed that some *HvFRF* genes identified in this study were significantly upregulated in response to drought stress (**Figure 6**), suggesting that some of these members play a role in drought resistance in barley.

### 4.2 The *FRF* gene in barley is evolutionarily specific

During long-term evolution, genes derived from the same ancestor (that is, the sequences of different members of the same gene family) become different, and each gene exhibits different expression regulation patterns and plays different functions (Mitchell-Olds and Schmitt, 2006). The study of gene families can help in understanding the evolutionary history of a class of genes, in determining whether there is a specific gene family in a species, and in discovering important functional genes (Thornton and DeSalle, 2000). FRF family members have only one FAR1 domain, which is considered to be a truncated FRS protein. It can compete with FRS for the binding site of target genes and play a regulatory role on target genes (Ma et al., 2016). At present, research on the FRF genes in crops is still very limited. In this study, Blast and hummer search identified various numbers of FRF genes from the genomes of dicotyledonous plants (soybean and grape). However, except for barley and brachypodium, FRF family genes were not found in other monocotyledonous crops (wheat, maize, rice, and sorghum). This demonstrated that FRF family genes are unique to barley and brachypodium in these monocotyledonous crops, suggesting that FRF family genes have specific roles in growth and development or stress resistance in barley. Meanwhile, in terms of evolution, the FRF genes of monocotyledonous plants (barley and brachypodium) and dicotyledonous plants (arabidopsis, soybean and grape) are on completely different branches (**Figure 1**). This indicated that the FRF genes of monocotyledonous and dicotyledonous plants are distantly related, and the FRF family members of barley and brachypodium are relatively more specific in evolution. Studies have reported that the main causes of gene family expansion include genome polyploidy, tandem duplication, fragment duplication, retrotransposition or exon duplication, and reorganization (Zhu et al., 2014; Bai et al., 2022). However, no collinearity gene pair was observed among the 22 *HvFRF* genes identified in this study (**Figure S1**), indicating that each member of the *HvFRF* gene family is relatively independent and may have different functions.

### 4.3 The promoter zone of FRF genes in barley contains abundant adversity-stress-response elements

Promoters are gene switches located in the upstream of the coding region of genes, which turn on and off the functional activity of genes and contain specific *cis*-elements. These elements are the binding targets of upstream regulatory genes, and they regulate the expression of target genes by binding to upstream genes (Hernandez-Garcia and Finer, 2014; Villao-Uzho et al., 2023). The analysis of *cis*-elements in the promoter region of *HvFRFs* revealed that the promoter region of *HvFRFs* contains a large number of hormone- and abiotic-stress-response elements. Significant differences were observed in element types and distribution characteristics between members of subfamilies I and II (**Figure 4**), indicating that *HvFRF* genes from subfamilies I and II may play different functions. The results revealed that *MaTIP1;2* gene promoter contains salicylic-acid-response element (TCA-element), which was introduced into *A. thaliana* to enhance drought and salt stress resistance (Song et al., 2018). Since various stresses can induce ABA synthesis, ABA is considered as a plant stress hormone (Yoon et al., 2020). Many genes that respond to ABA signals contain ABRE elements in the promoter zones, and these genes often play a role in the response of plants to abiotic stress (Dinneny et al., 2008). For example, the *DREB2A* gene in arabidopsis has the function of drought resistance (Kim et al., 2011), and the *OsDREB* gene in rice responds to drought, high-salt, and low-temperature stresses (Dubouzet et al., 2003). The promoter zones of drought- and salt-tolerance genes in arabidopsis contain a certain amount of TCA-element, ABRE, gibatellin-responsive element (GARE motif), methyl jasmonate response element (MeJA-element), and drought-response element (MBS) (Shariatipour and Heidari, 2018). In this study, the promoter zone of *HvFRF* genes in subfamily I contained a large amount of ABRE and MeJA-element, whereas that in subfamily II contained more TCA-element and GARE motifs. Additionally, the promoter region of some members contained MBS (**Figure 4**). Thus, it is speculated that *HvFRF* genes bind to upstream regulators through cis-elements in the promoter zone and play a role in abiotic stress resistance.

### 4.4 *HvFRF9* plays an important role in drought resistance

The results of tissue expression profiling revealed that the expression of *HvFRF9* in various tissues of barley was obviously higher than other *HvFRFs* (**Figure 5**). To identify the FRF genes related to drought resistance in barley, the expression characteristics of *HvFRF* genes in the same evolutionary branch as *HvFRF9* in response to drought stress were analyzed. The results revealed that *HvFRF9* gene responded most significantly to drought stress (**Figure 6**), suggesting that this gene was involved in the regulation of drought resistance in barley. The epidermal cells of the root are in direct contact with the soil and are the main parts that absorb water and nutrients. In addition, the epidermal cells can form root hair increasing the surface area of the root system, which is conducive to the absorption of water and nutrients (He et al., 2015). Compared with *in-situ* hybridization, *in-situ* PCR is a relatively simple and rapid method for detecting the location of gene expression (Athman et al., 2014). The results of *in-situ* PCR revealed that *HvFRF9* was highly expressed in the epidermal cells of barley root (**Figure 7A**), indicating that this gene is beneficial for the absorption of water or nutrients by roots. Xylem and phloem are responsible for transporting water absorbed by roots and ions dissolved in water, making them available for other tissues or organs; additionally, they provide support to plant body (Huang et al., 2018). *HvFRF9* was mainly expressed in the xylem and phloem of barley roots and leaves (**Figure 7A**), indicating that this gene also plays a role in water or nutrient transport.

Drought stress can decrease the relative water content in the leaves, resulting in leaf wilting and stem tip bending, which affects the normal growth of plants (Fang and Xiong, 2015). Meanwhile, drought stress can lead to excessive ROS accumulation in plants, causing oxidative damage to plants (Lee and Park, 2012). Additionally, MDA content in plants increased after drought stress, resulting in cytoplasmic membrane damage (Ma et al., 2015). In this study, under drought stress, the degree of loss of green color, wilting, O_2_^−^ accumulation, and MDA content were lower in the leaves of *HvFRF9*-overexpressing arabidopsis than in those of wild-type plants (**Figures 8**, **9**). This suggested that overexpression of *HvFRF9* improved the ability of arabidopsis to resist drought. Osmotic regulation is an important mechanism for plants to resist drought stress. Plants can adapt to the external environment by regulating the osmotic potential of cells (Dubois and Inzé, 2020). PRO is an important osmoregulatory organic substance, and increase in its content under drought conditions helps to prevent cell or tissue dehydration (Wang et al., 2022). In the process of resisting stress, plants have evolved a complex antioxidant system to reduce the production of excessive ROS and avoid serious oxidative damage to plants. It mainly includes SOD, POD, CAT, and other antioxidant enzymes (Ahmadi et al., 2022). SOD, as the first line of antioxidant defense, converts O_2_^−^ into H_2_O_2_ and O_2_ through the dismutation reaction, and CAT and POD further convert toxic endogenous H_2_O_2_ into H_2_O and O_2_ (Anjum et al., 2012). Under drought stress, the increase in PRO content and SOD, CAT, and POD activities were significantly higher in *HvFRF9*-overexpressing arabidopsis than in wild-type plants (**Figure 9**). These results indicated that *HvFRF9* could reduce the osmotic stress and increase the antioxidant ability of the plants by increasing the PRO content and antioxidant enzyme activities, thus enhancing the drought resistance of the plants.

In conclusion, drought is one of the most dominant abiotic stresses affecting growth and yield of crops. FRF genes are novel transcription factors unique in plants. They play a role in stress resistance in plants. With wide adaptability and high stress-resistance, barley is an excellent candidate for the identification of stress resistance genes. However, studies on the FRF genes in barley are few. In this study, 22 *HvFRFs* were identified using genome-wide analysis, and their gene sequence, gene structure, evolutionary relationship, chromosome localization, *cis*-element in promoter, collinearity, tissue expression site, and expression profile in response to drought stress were analyzed. *HvFRF9* with significantly upregulated expression in response to drought stress was identified. Under drought stress, leaf chlorosis and wilting and MDA and O_2_^−^ contents were significantly lower and fresh weight; root length; PRO content; and SOD, CAT, and POD activities were significantly higher in *HvFRF9*-overexpressed arabidopsis than in wild-type plants. This suggested that *HvFRF9* overexpression could enhance drought resistance in arabidopsis. Therefore, *HvFRF9* exhibits drought resistance function. This study enhanced the understanding of drought resistance in plants and provided candidate genes for genetic improvement of drought resistance in barley or other plants.

## Data availability statement

The original contributions presented in the study are included in the article/**Supplementary Material**. Further inquiries can be directed to the corresponding author.

## Author contributions

XH: Conceptualization, Data curation, Funding acquisition, Investigation, Supervision, Visualization, Writing – original draft, Writing – review & editing. YH: Data curation, Formal analysis, Supervision, Writing – review & editing. YD: Conceptualization, Data curation, Formal analysis, Investigation, Writing – original draft. YG: Data curation, Writing – original draft. XS: Data curation, Writing – original draft. WC: Data curation, Validation, Writing – original draft. XX: Validation, Writing – original draft. CS: Validation, Writing – original draft. YL: Validation, Writing – original draft. BR: Validation, Writing – original draft. HY: Validation, Writing – original draft. JZ: Funding acquisition, Supervision, Writing – review & editing. WM: Supervision, Writing – review & editing. PM: Conceptualization, Funding acquisition, Supervision, Writing – review & editing.

## Supplementary material

Supplementary Figure 1 Gene duplication events in *HvFRFs* in barley. From outside to inside are the schematic diagram of the seven chromosomes in barley, peak map of gene density on barley chromosomes, and scale of gene density on barley chromosome, with red and blue representing the regions of high and low gene densities, respectively.

Supplementary Figure 2 Cloning results of *HvFRF9.* **(A)** Agarose gel electrophoresis of *HvFRF9* cDNA and DNA. (B) Nucleotide and amino acid sequences of the ORF region of *HvFRF9*. M: DL2000 DNA marker; 1: cDNA amplification bands of *HvFRF9*; 2: DNA amplification bands of *HvFRF9*.

Supplementary Figure 3 Amino acid sequence alignment analysis of HvFRF9 with four FRF in arabidopsis. **(A)** Amino acid sequences; the part marked in red is the FAR1 conserved domain. **(B)** HMM logo plot of the conserved domain of FAR1, with larger letters indicating more conserved amino acids.

Supplementary Figure 4 Structural prediction of *HvFRF9* gene. **(A)** The secondary structure of *HvFRF9*. **(B)** The 3D model of *HvFRF9*.

## References

[B1] Aguilar-Martínez J. A. Uchida N. Townsley B. West D. A. Yanez A. Lynn N.. (2015). Transcriptional, posttranscriptional, and posttranslational regulation of *SHOOT MERISTEMLESS* gene expression in Arabidopsis determines gene function in the shoot apex. Plant Physiol. 167, 424–442. doi: 10.1104/pp.114.248625 25524441 PMC4326739

[B2] Ahmadi H. Abbasi A. Taleei A. Mohammadi V. Pueyo J. J. (2022). Antioxidant response and calcium-dependent protein kinases involvement in canola (*Brassica napus* L.) tolerance to drought. Agronomy-Basel 12, 125. doi: 10.3390/agronomy12010125

[B3] Anjum S. A. Farooq M. Xie X.-Y. Liu X.-J. Ijaz M. F. (2012). Antioxidant defense system and proline accumulation enables hot pepper to perform better under drought. Scientia Hortic. 140, 66–73. doi: 10.1016/j.scienta.2012.03.028

[B4] Athman A. Tanz S. K. Conn V. M. Jordans C. Mayo G. M. Ng W. W.. (2014). Protocol: a fast and simple in *situ* PCR method for localising gene expression in plant tissue. Plant Methods 10, 29. doi: 10.1186/1746-4811-10-29 25250056 PMC4171716

[B5] Bai J. Song M. J. Gao J. Li G. (2022). Whole genome duplication and dispersed duplication characterize the evolution of the plant PINOID gene family across plant species. Gene. 829, 146494. doi: 10.1016/j.gene.2022.146494 35447241

[B6] Bournonville C. F. G. Diaz-Ricci J. C. (2011). Quantitative determination of superoxide in plant leaves using a modified NBT staining method. Phytochem. Analysis. 22, 268–271. doi: 10.1002/pca.1275 21360621

[B7] Carter A. Y. Hawes M. C. Ottman M. J. (2019). Drought-tolerant barley: I. Field observations of growth and development. Agronomy-Basel. 9, 221. doi: 10.3390/agronomy9050221

[B8] Chen C. Chen H. Zhang Y. Thomas H. R. Frank M. H. He Y.. (2020). TBtools: an integrative toolkit developed for interactive analyses of big biological data. Mol. Plant 13, 1194–1202. doi: 10.1016/j.molp.2020.06.009 32585190

[B9] Chen K. Li G.-J. Bressan R. A. Song C.-P. Zhu J.-K. Zhao Y. (2020). Abscisic acid dynamics, signaling, and functions in plants. J. Integr. Plant Biol. 62, 25–54. doi: 10.1111/jipb.12899 31850654

[B10] Dai J. Sun J. Peng W. Liao W. Zhou Y. Zhou X.-R.. (2022). FAR1/FHY3 transcription factors positively regulate the salt and temperature stress responses in Eucalyptus grandis. Front. Plant Sci. 13, 883654. doi: 10.3389/fpls.2022.883654 35599891 PMC9115564

[B11] Dinneny J. R. Long T. A. Wang J. Y. Jung J. W. Mace D. Pointer S.. (2008). Cell Identity Mediates the response of Arabidopsis Roots to abiotic stress. Science. 320, 942–945. doi: 10.1126/science.1153795 18436742

[B12] Dubois M. Inzé D. (2020). Plant growth under suboptimal water conditions: early responses and methods to study them. J. Exp. Bot. 71, 1706–1722. doi: 10.1093/jxb/eraa037 31967643

[B13] Dubouzet J. G. Sakuma Y. Ito Y. Kasuga M. Dubouzet E. G. Miura S.. (2003). *OsDREB* genes in rice, *Oryza sativa* L., encode transcription activators that function in drought-, high-salt- and cold-responsive gene expression. Plant J. 33, 751–763. doi: 10.1046/j.1365-313X.2003.01661.x 12609047

[B14] Fang Y. Xiong L. (2015). General mechanisms of drought response and their application in drought resistance improvement in plants. Cell Mol. Life Sci. 72, 673–689. doi: 10.1007/s00018-014-1767-0 25336153 PMC11113132

[B15] Fernández-Calvo P. Iñigo S. Glauser G. Vanden Bossche R. Tang M. Li B.. (2020). FRS7 and FRS12 recruit NINJA to regulate expression of glucosinolate biosynthesis genes. New Phytol. 227, 1124–1137. doi: 10.1111/nph.16586 32266972

[B16] He X. Zeng J. Cao F. Ahmed I. M. Zhang G. Vincze E.. (2015). *HvEXPB7*, a novel β-expansin gene revealed by the root hair transcriptome of Tibetan wild barley, improves root hair growth under drought stress. J. Exp. Bot. 66, 7405–7419. doi: 10.1093/jxb/erv436 26417018 PMC4765802

[B17] Hernandez-Garcia C. M. Finer J. J. (2014). Identification and validation of promoters and *cis*-acting regulatory elements. Plant Sci. 217, 109–119. doi: 10.1016/j.plantsci.2013.12.007 24467902

[B18] Huang C.-W. Domec J.-C. Palmroth S. Pockman W. T. Litvak M. E. Katul G. G. (2018). Transport in a coordinated soil-root-xylem-phloem leaf system. Adv. Water Resour. 119, 1–16. doi: 10.1016/j.advwatres.2018.06.002

[B19] Hudson M. Ringli C. Boylan M. T. Quail P. H. (1999). The FAR1 locus encodes a novel nuclear protein specific to phytochrome A signaling. Genes Dev. 13, 2017–2027. doi: 10.1101/gad.13.15.2017 10444599 PMC316922

[B20] Joly-Lopez Z. Hoen D. R. Blanchette M. Bureau T. E. (2016). Phylogenetic and genomic analyses resolve the origin of important plant genes derived from transposable elements. Mol. Biol. Evol. 33, 1937–1956. doi: 10.1093/molbev/msw067 27189548 PMC4948706

[B21] Kebede A. Kang M. S. Bekele E. (2019). Advances in mechanisms of drought tolerance in crops, with emphasis on barley. Adv. Agron. 156, 265–314. doi: 10.1016/bs.agron.2019.01.008

[B22] Kim J.-S. Mizoi J. Yoshida T. Fujita Y. Nakajima J. Ohori T.. (2011). An ABRE promoter sequence is involved in osmotic stress-responsive expression of the *DREB2A* gene, which encodes a transcription factor regulating drought-inducible genes in Arabidopsis. Plant Cell Physiol. 52, 2136–2146. doi: 10.1093/pcp/pcr143 22025559

[B23] Kiseleva A. A. Potokina E. K. Salina E. A. (2017). Features of *Ppd-B1* expression regulation and their impact on the flowering time of wheat near-isogenic lines. BMC Plant Biol. 17, 172. doi: 10.1186/s12870-017-1126-z 29143607 PMC5688470

[B24] Lee S. Park C.-M. (2012). Regulation of reactive oxygen species generation under drought conditions in Arabidopsis. Plant Signal Behav. 7, 599–601. doi: 10.4161/psb.19940 22580707 PMC3442848

[B25] Li D. Fu X. Guo L. Huang Z. Li Y. Liu Y.. (2016). *FAR-RED ELONGATED HYPOCOTYL3* activates *SEPALLATA2* but inhibits *CLAVATA3* to regulate meristem determinacy and maintenance in Arabidopsis. P Natl. Acid Sci. U.S.A. 113, 9375–9380. doi: 10.1073/pnas.1602960113 PMC4995929 27469166

[B26] Li G. Siddiqui H. Teng Y. Lin R. Wan X.-Y. Li J.. (2011). Coordinated transcriptional regulation underlying the circadian clock in Arabidopsis. Nat. Cell Biol. 13, 616–622. doi: 10.1038/ncb2219 21499259

[B27] Lin R. Ding L. Casola C. Ripoll D. R. Feschotte C. Wang H. (2007). Transposase-derived transcription factors regulate light signaling in Arabidopsis. Science. 318, 1302–1305. doi: 10.1126/science.1146281 18033885 PMC2151751

[B28] Lin R. Wang H. (2004). Arabidopsis *FHY3/FAR1* gene family and distinct roles of its members in light control of Arabidopsis development. Plant Physiol. 136, 4010–4022. doi: 10.1104/pp.104.052191 15591448 PMC535833

[B29] Liu Y. Wei H. Ma M. Li Q. Kong D. Sun J.. (2019). Arabidopsis FHY3 and FAR1 regulate the balance between growth and defense responses under shade conditions. Plant Cell. 31, 2089–2106. doi: 10.1105/tpc.18.00991 31311834 PMC6751128

[B30] Liu Y. Xie Y. Wang H. Ma X. Yao W. Wang H. (2017). Light and ethylene coordinately regulate the phosphate starvation response through transcriptional regulation of PHOSPHATE STARVATION RESPONSE1. Plant Cell. 29, 2269–2284. doi: 10.1105/tpc.17.00268 28842534 PMC5635990

[B31] Ma J. Du G. Li X. Zhang C. Guo J. (2015). A major locus controlling malondialdehyde content under water stress is associated with *Fusarium* crown rot resistance in wheat. Mol. Genet. Genomics 290, 1955–1962. doi: 10.1007/s00438-015-1053-3 25939503

[B32] Ma L. Li G. (2018). FAR1-RELATED SEQUENCE (FRS) and FRS-RELATED FACTOR (FRF) family proteins in Arabidopsis growth and development. Front. Plant Sci. 9, 692. doi: 10.3389/fpls.2018.00692 29930561 PMC6000157

[B33] Ma L. Li G. (2021). Arabidopsis FAR-RED ELONGATED HYPOCOTYL3 negatively regulates carbon starvation responses. Plant Cell Environ. 44, 1816–1829. doi: 10.1111/pce.14044 33715163

[B34] Ma L. Tian T. Lin R. Deng X.-W. Wang H. Li G. (2016). *Arabidopsis* FHY3 and FAR1 regulate light-induced *myo* -inositol biosynthesis and oxidative stress responses by transcriptional activation of MIPS1. Mol. Plant 9, 541–557. doi: 10.1016/j.molp.2015.12.013 26714049

[B35] Ma L. Xue N. Fu X. Zhang H. Li G. (2017). *Arabidopsis thaliana* FAR-RED ELONGATED HYPOCOTYLS3 (FHY3) and FAR-RED-IMPAIRED RESPONSE1 (FAR1) modulate starch synthesis in response to light and sugar. New Phytol. 213, 1682–1696. doi: 10.1111/nph.14300 27859295

[B36] Mitchell-Olds T. Schmitt J. (2006). Genetic mechanisms and evolutionary significance of natural variation in Arabidopsis. Nature. 441, 947–952. doi: 10.1038/nature04878 16791187

[B37] Ouyang X. Li J. Li G. Li B. Chen B. Shen H.. (2011). Genome-wide binding site analysis of FAR-RED ELONGATED HYPOCOTYL3 reveals its novel function in Arabidopsis development. Plant Cell. 23, 2514–2535. doi: 10.1105/tpc.111.085126 21803941 PMC3226222

[B38] Salojärvi J. Smolander O.-P. Nieminen K. Rajaraman S. Safronov O. Safdari P.. (2017). Genome sequencing and population genomic analyses provide insights into the adaptive landscape of silver birch. Nat. Genet. 49, 904–912. doi: 10.1038/ng.3862 28481341

[B39] Shariatipour N. Heidari B. (2018). Investigation of drought and salinity tolerance related genes and their regulatory mechanisms in Arabidopsis (Arabidopsis thaliana). Open Bioinf. J. 11, 12–28. doi: 10.2174/1875036201811010012

[B40] Song S. Xu Y. Huang D. Miao H. Liu J. Jia C.. (2018). Identification of a novel promoter from banana aquaporin family gene (*MaTIP1*;*2*) which responses to drought and salt-stress in transgenic Arabidopsis thaliana. Plant Physiol. Bioch 128, 163–169. doi: 10.1016/j.plaphy.2018.05.003 29778840

[B41] Stirnberg P. Zhao S. Williamson L. Ward S. Leyser O. (2012). FHY3 promotes shoot branching and stress tolerance in Arabidopsis in an AXR1-dependent manner. Plant J. 71, 907–920. doi: 10.1111/j.1365-313X.2012.05038.x 22540368

[B42] Tang W. Ji Q. Huang Y. Jiang Z. Bao M. Wang H.. (2013). FAR-RED ELONGATED HYPOCOTYL3 and FAR-RED IMPAIRED RESPONSE1 transcription factors integrate light and abscisic acid signaling in Arabidopsis. Plant Physiol. 163, 857–866. doi: 10.1104/pp.113.224386 23946351 PMC3793063

[B43] Thornton J. W. DeSalle R. (2000). Gene family evolution and homology: genomics meets phylogenetics. Annu. Rev. Genom Hum. G. 1, 41–73. doi: 10.1146/annurev.genom.1.1.41 11701624

[B44] Villao-Uzho L. Chávez-Navarrete T. Pacheco-Coello R. Sánchez-Timm E. Santos-Ordóñez E. (2023). Plant promoters: their identification, characterization, and role in gene regulation. Genes. 14, 1226. doi: 10.3390/genes14061226 37372407 PMC10298551

[B45] Wang H. Deng X. W. (2002). Arabidopsis FHY3 defines a key phytochrome A signaling component directly interacting with its homologous partner FAR1. EMBO J. 21, 1339–1349. doi: 10.1093/emboj/21.6.1339 11889039 PMC125923

[B46] Wang T. Ren L. Li C. Zhang D. Zhang X. Zhou G.. (2021). The genome of a wild *Medicago* species provides insights into the tolerant mechanisms of legume forage to environmental stress. BMC Biol. 19, 96. doi: 10.1186/s12915-021-01033-0 33957908 PMC8103640

[B47] Wang W. Tang W. Ma T. Niu D. Jin J. B. Wang H.. (2016). A pair of light signaling factors FHY3 and FAR1 regulates plant immunity by modulating chlorophyll biosynthesis. J. Integr. Plant Biol. 58, 91–103. doi: 10.1111/jipb.12369 25989254 PMC4736690

[B48] Wang Z. Yang Y. Yadav V. Zhao W. He Y. Zhang X.. (2022). Drought-induced proline is mainly synthesized in leaves and to roots in watermelon under water deficit. Hortic. Plant J. 8, 615–626. doi: 10.1016/j.hpj.2022.06.009

[B49] Xu D. Wu D. Li X.-H. Jiang Y. Tian T. Chen Q.. (2020). Light and abscisic acid coordinately regulate greening of seedlings. J. Plant Physiol. 183, 1281–1294. doi: 10.1104/pp.20.00503 PMC7333693 32414897

[B50] Yoder J. B. Briskine R. Mudge J. Farmer A. Paape T. Steele K.. (2013). Phylogenetic signal variation in the genomes of *Medicago* (Fabaceae). Syst. Biol. 62, 424–438. doi: 10.1093/sysbio/syt009 23417680

[B51] Yoon Y. Seo D. H. Shin H. Kim H. J. Kim C. M. Jang G. (2020). The role of stress-responsive transcription factors in modulating abiotic stress tolerance in plants. Agronomy-Basel. 10, 788. doi: 10.3390/agronomy10060788

[B52] Zhang S. Xu F. Zhang Y. Lin J. Song C. Fang X. (2015). Fine mapping and candidate gene analysis of a novel *PANICLE AND SPIKELET DEGENERATION* gene in rice. Euphytica. 206, 793–803. doi: 10.1007/s10681-015-1525-x

[B53] Zhu Y. Wu N. Song W. Yin G. Qin Y. Yan Y.. (2014). Soybean (*Glycine max*) expansin gene superfamily origins: segmental and tandem duplication events followed by divergent selection among subfamilies. BMC Plant Biol. 14, 93. doi: 10.1186/1471-2229-14-93 24720629 PMC4021193

