# Supplementary figures and images for: Genome-wide analysis of FRF gene family and functional identification of HvFRF9 under drought stress in barley
Source: Front Plant Sci. 2024 Jan 24;15:1347842. doi: 10.3389/fpls.2024.1347842 (PMC10847358; doi:10.3389/fpls.2024.1347842)

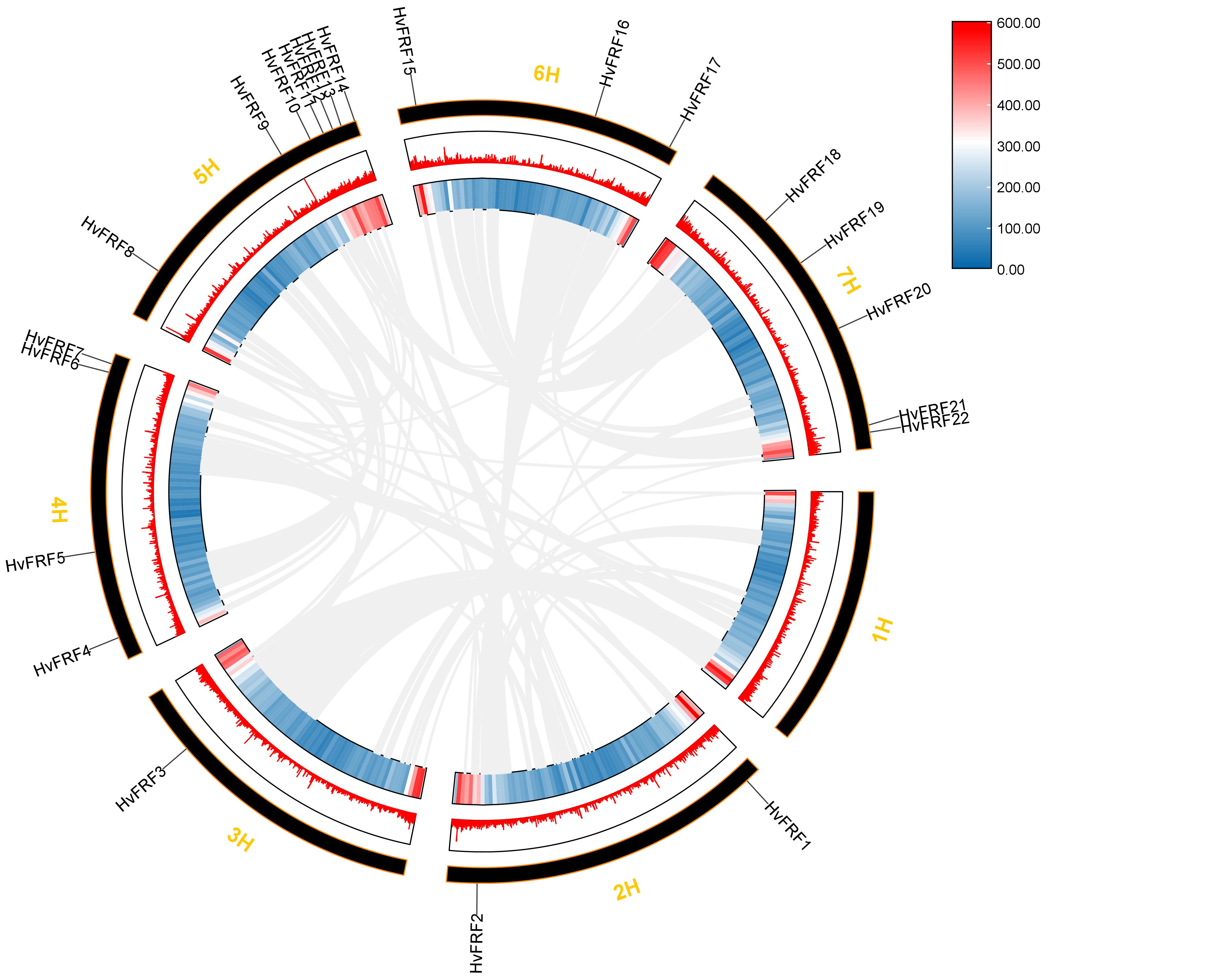

Supplement: Supplementary Figure 1 — Gene duplication events in HvFRFs in barley. From outside to inside are the schematic diagram of the seven chromosomes in barley, peak map of gene density on barley chromosomes, and scale of gene density on barley chromosome, with red and blue representing the regions of high and low gene densities, respectively. [file Image_1.jpeg]

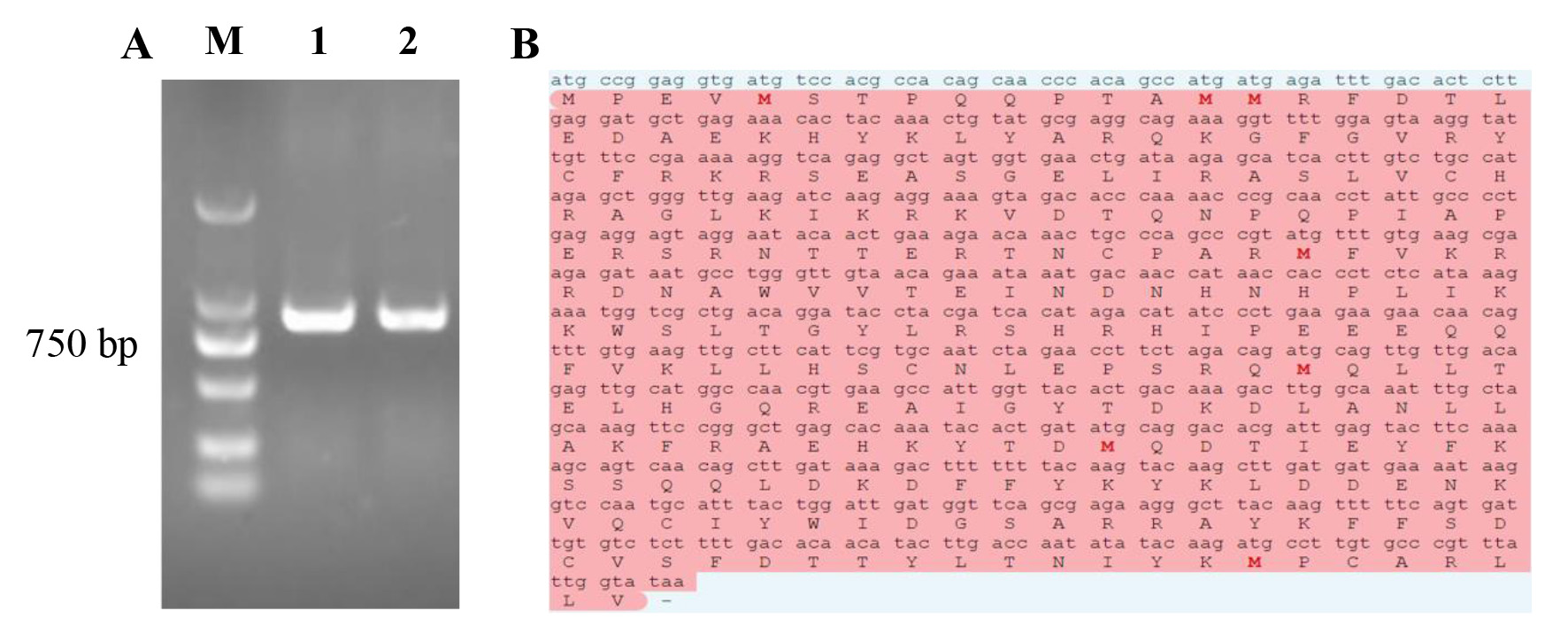

Supplement: Supplementary Figure 2 — Cloning results of HvFRF9. (A) Agarose gel electrophoresis of HvFRF9 cDNA and DNA. (B) Nucleotide and amino acid sequences of the ORF region of HvFRF9. M: DL2000 DNA marker; 1: cDNA amplification bands of HvFRF9; 2: DNA amplification bands of HvFRF9. [file Image_2.jpeg]

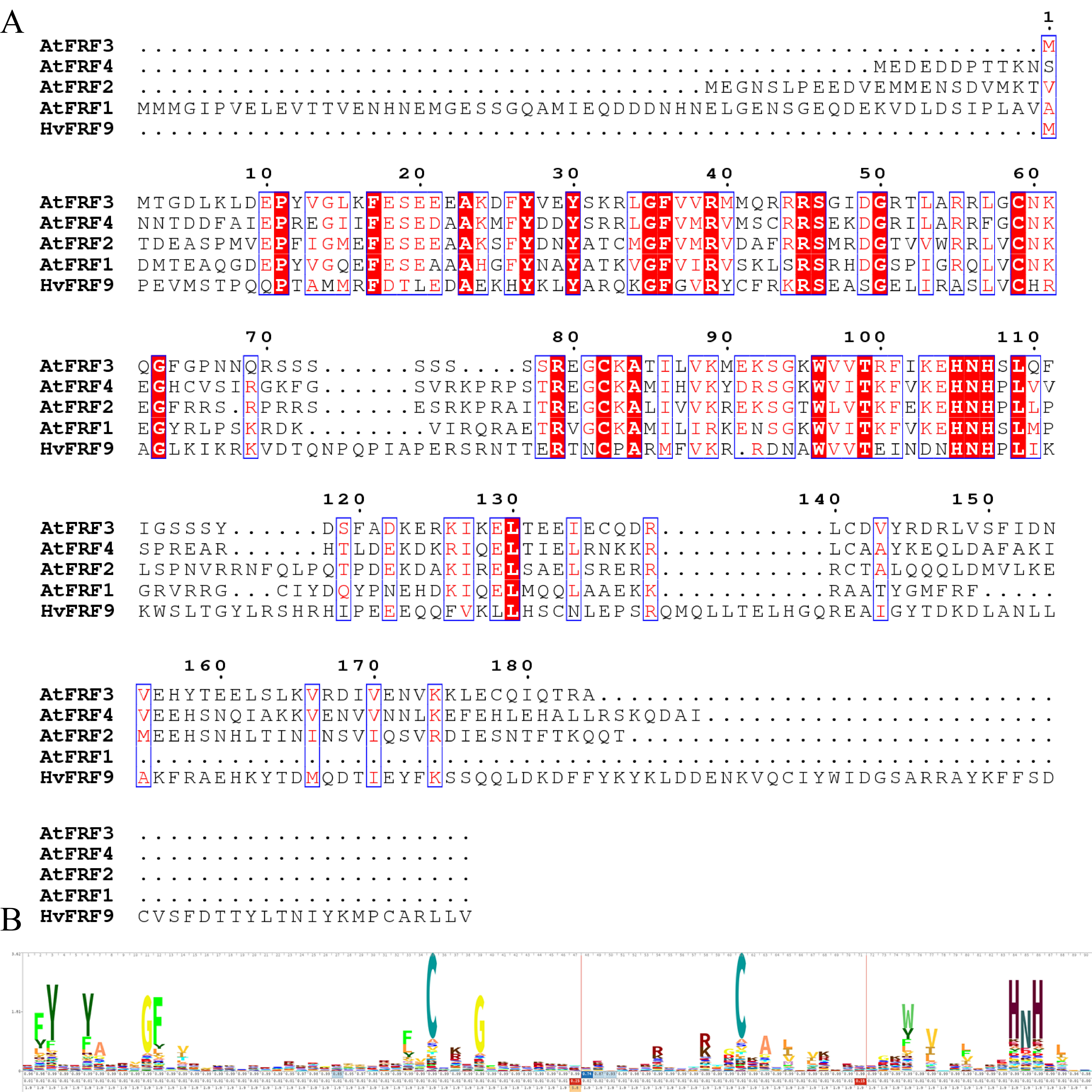

Supplement: Supplementary Figure 3 — Amino acid sequence alignment analysis of HvFRF9 with four FRF in arabidopsis. (A) Amino acid sequences; the part marked in red is the FAR1 conserved domain. (B) HMM logo plot of the conserved domain of FAR1, with larger letters indicating more conserved amino acids. [file Image_3.jpeg]

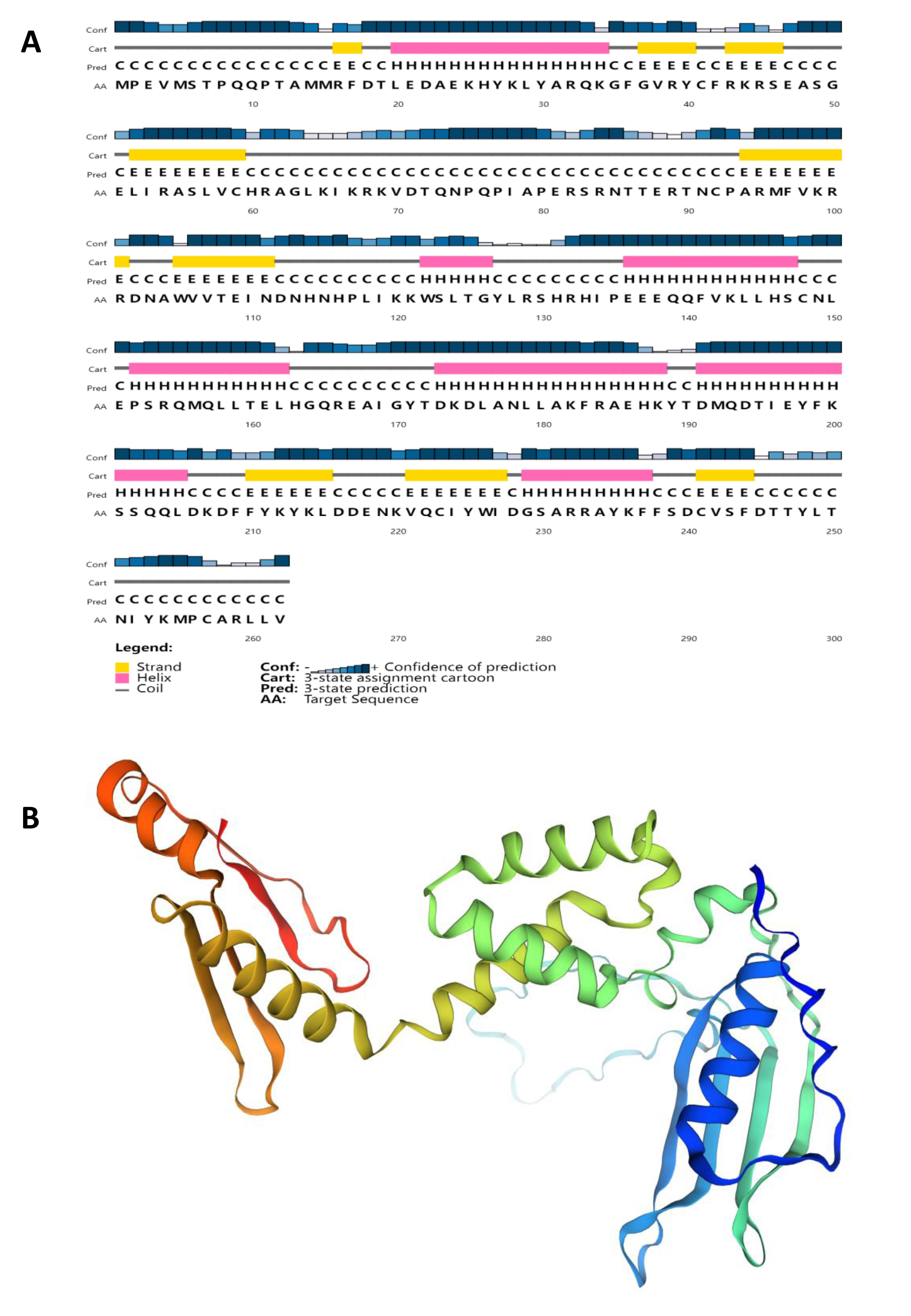

Supplement: Supplementary Figure 4 — Structural prediction of HvFRF9 gene. (A) The secondary structure of HvFRF9. (B) The 3D model of HvFRF9. [file Image_4.jpeg]
